# Supplementary material for: Isolated copper single sites for high-performance electroreduction of carbon monoxide to multicarbon products
Source: Nat Commun. 2021 Jan 11;12:238. doi: 10.1038/s41467-020-20336-4 (PMC7801608; doi:10.1038/s41467-020-20336-4)
Supplement: Supplementary file 1 — Supplementary Information [file 41467_2020_20336_MOESM1_ESM.pdf]

Supplementary Information for

Isolated copper single sites for high-performance electroreduction of carbon monoxide to multicarbon products

Bao et al.

Supplementary Figures

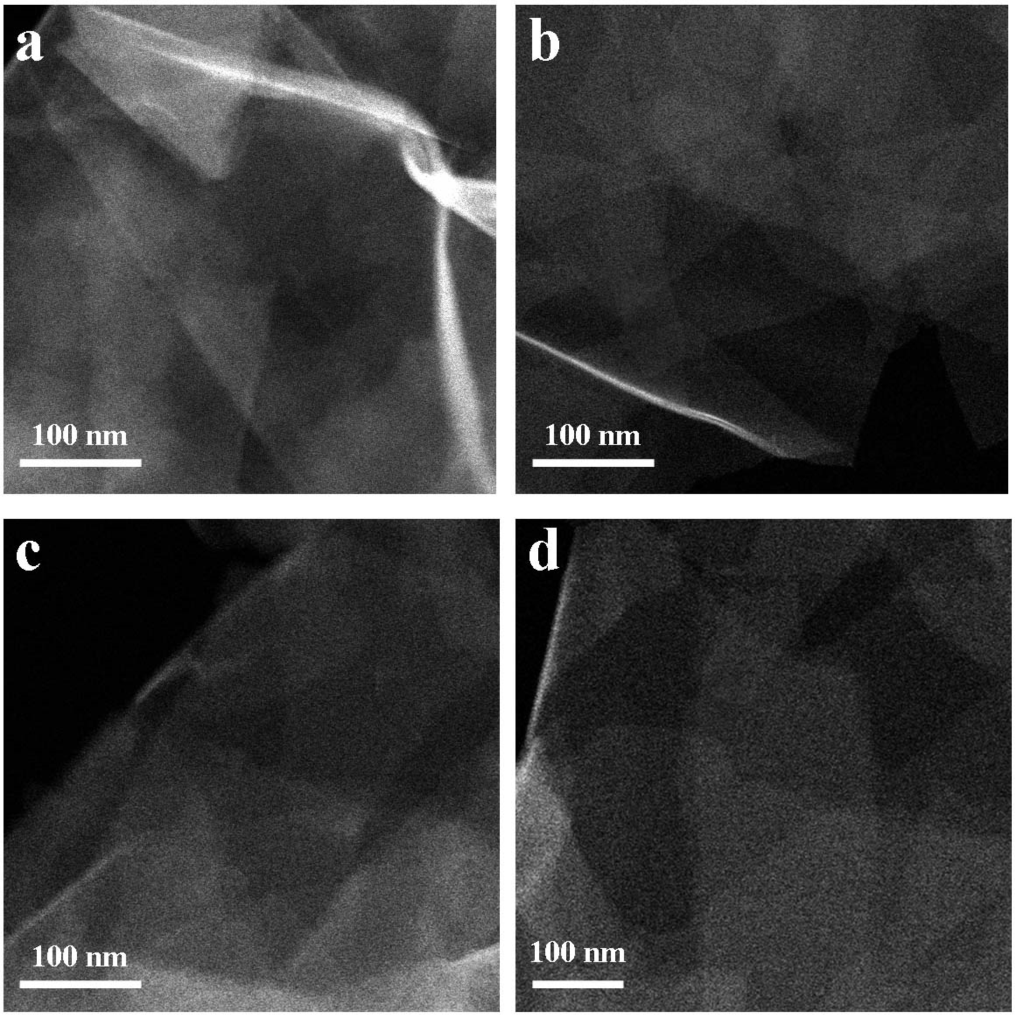

Supplementary Fig. 1. HAADF-STEM image of Cu-SA/$Ti_3C_2T_x$. It shows a nanosheet morphology, and no Cu nanoclusters or nanoparticles can be observed.

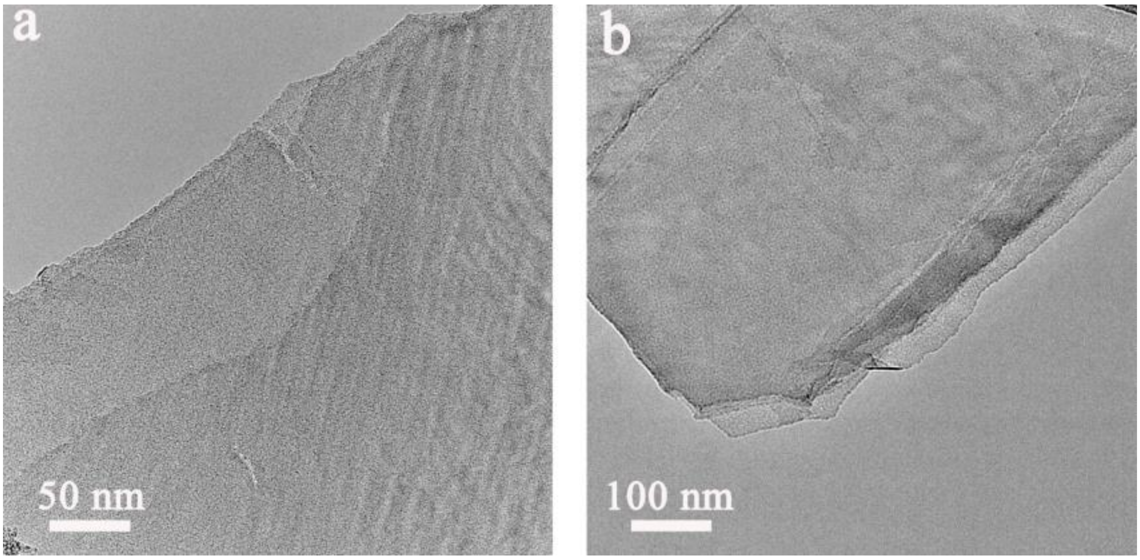

Supplementary Fig. 2. Additional TEM images of Cu-SA/$Ti_3C_2T_x$. Clearly, no Cu nanoparticles were observed.

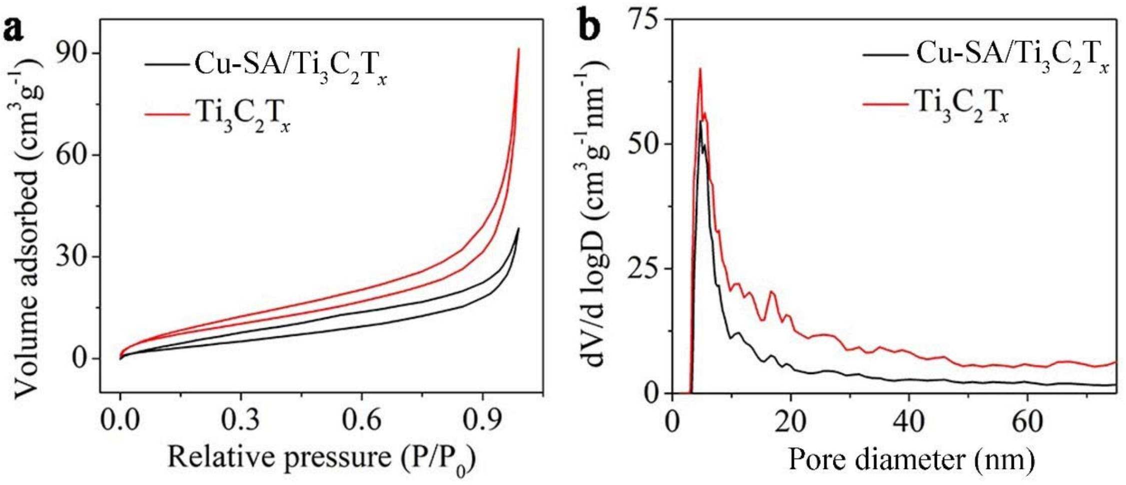

Supplementary Fig. 3. The N_2 physical adsorption results. (a) N_2 adsorption/desorption isotherm curves, and (b) the pore size distribution of the as-prepared Cu-SA/$Ti_3C_2T_x$ and $Ti_3C_2T_x$ samples calculated by an NLDFT method. It indicates the presence of mesopores of about 5–10 nm.

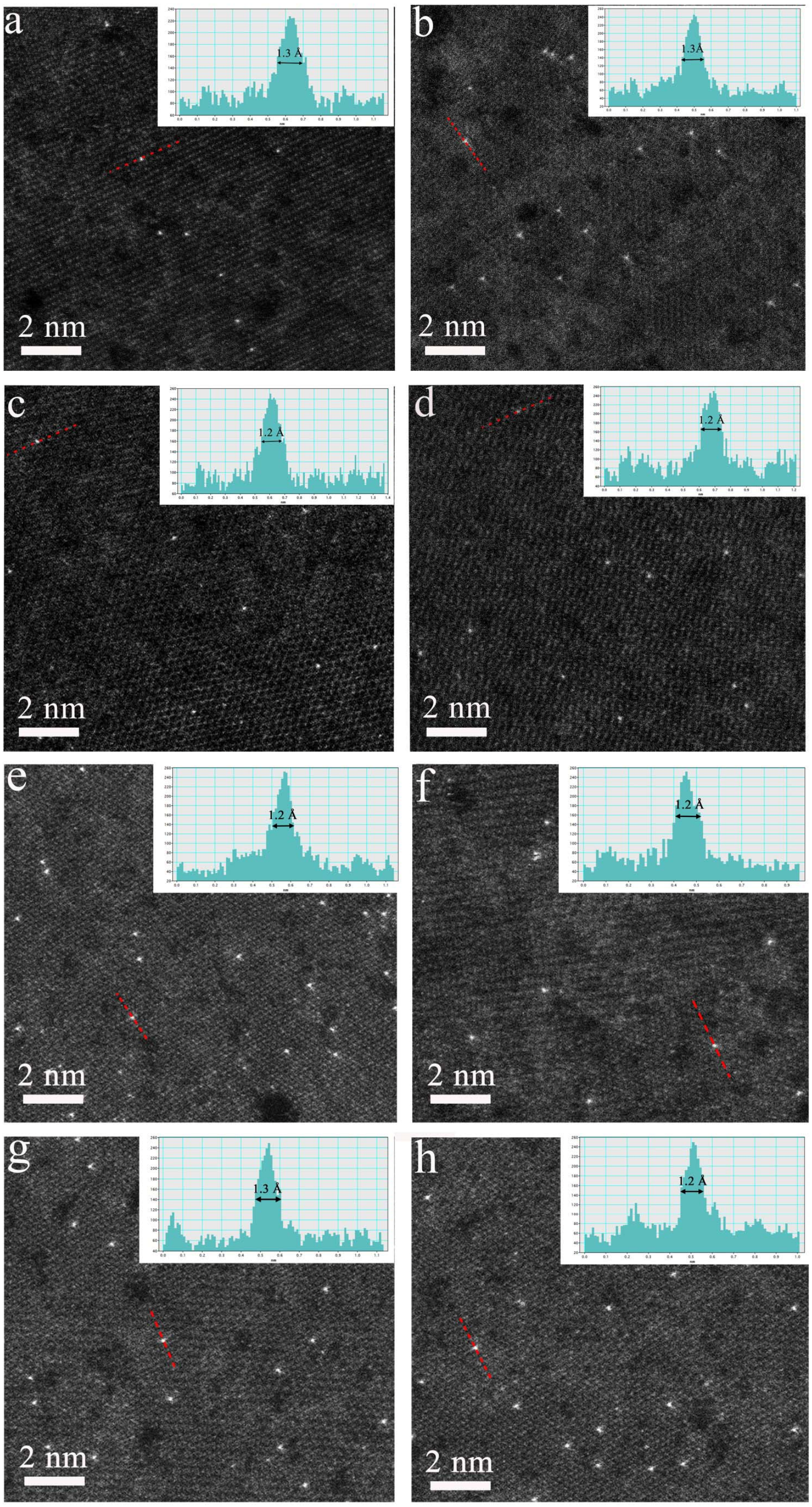

Supplementary Fig. 4. The additional atomic-resolution HAADF-STEM images of the as-prepared Cu-SA/$Ti_3C_2T_x$ sample, which were taken from random positions. Inserts are the HAADF intensity profile of a single Cu atom in (a-h), showing an intensity profile (0.01 nm/pixel). The full width at half maximum values of each peak in line profiles is *ca*. 1.2 Å, which is close to the radius of the Cu atoms (1.4 Å, reported in https://periodic.lanl.gov/29.shtml), proving the existence of isolated Cu atoms.

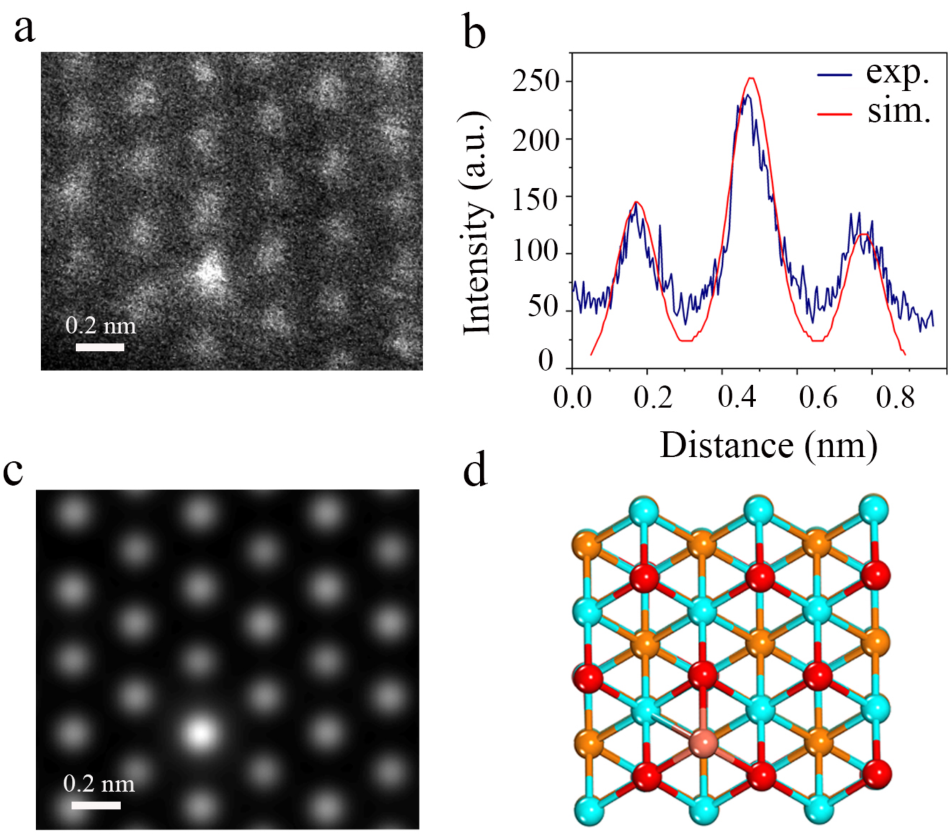

Supplementary Fig. 5. The simulation of the atomic resolution HAADF-STEM image of Cu-SA/$Ti_3C_2T_x$. (a, c) The experimental atomic resolution HAADF-STEM image (a) and the corresponding simulated image (c). (b) The intensity of the experimental HAADF-STEM and the corresponding simulated images. (d) The DFT-optimized Cu-SA/$Ti_3C_2T_x$ structure. The frozen phone approximated multislice simulations were conducted with the QSTEM program. The intensity of the simulated image matched well with the experimental ones. Despite the atomic number (Z), the sample thickness, defocus values, electron channeling effects, the spacial position of atoms, debye-waller factors, cross-talk effects and so on, also influence the intensities of the HAADF-STEM images. For Cu-SA/$Ti_3C_2T_x$, the well-arrangement of Ti atoms, 2-dimensional ultrathin structure and the flat surface of $Ti_3C_2T_x$ all lead to a better view for the attached Cu atoms.

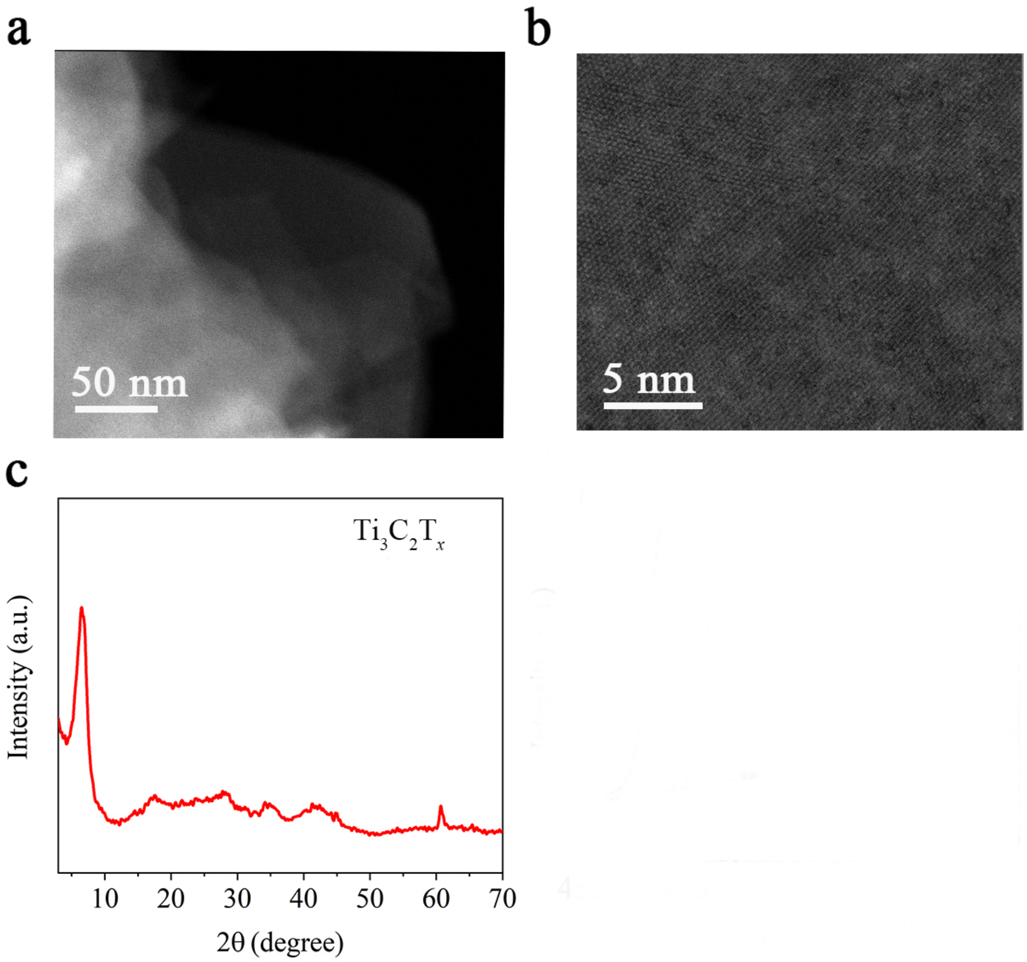

Supplementary Fig. 6. Characterizations of pure $Ti_3C_2T_x$. (a) HAADF-STEM image, (b) Atomic-resolution HAADF-STEM image and (c) XRD pattern. A homogenous contrast without sharp bright spots can be observed in the $Ti_3C_2T_x$ substrate in **b**.

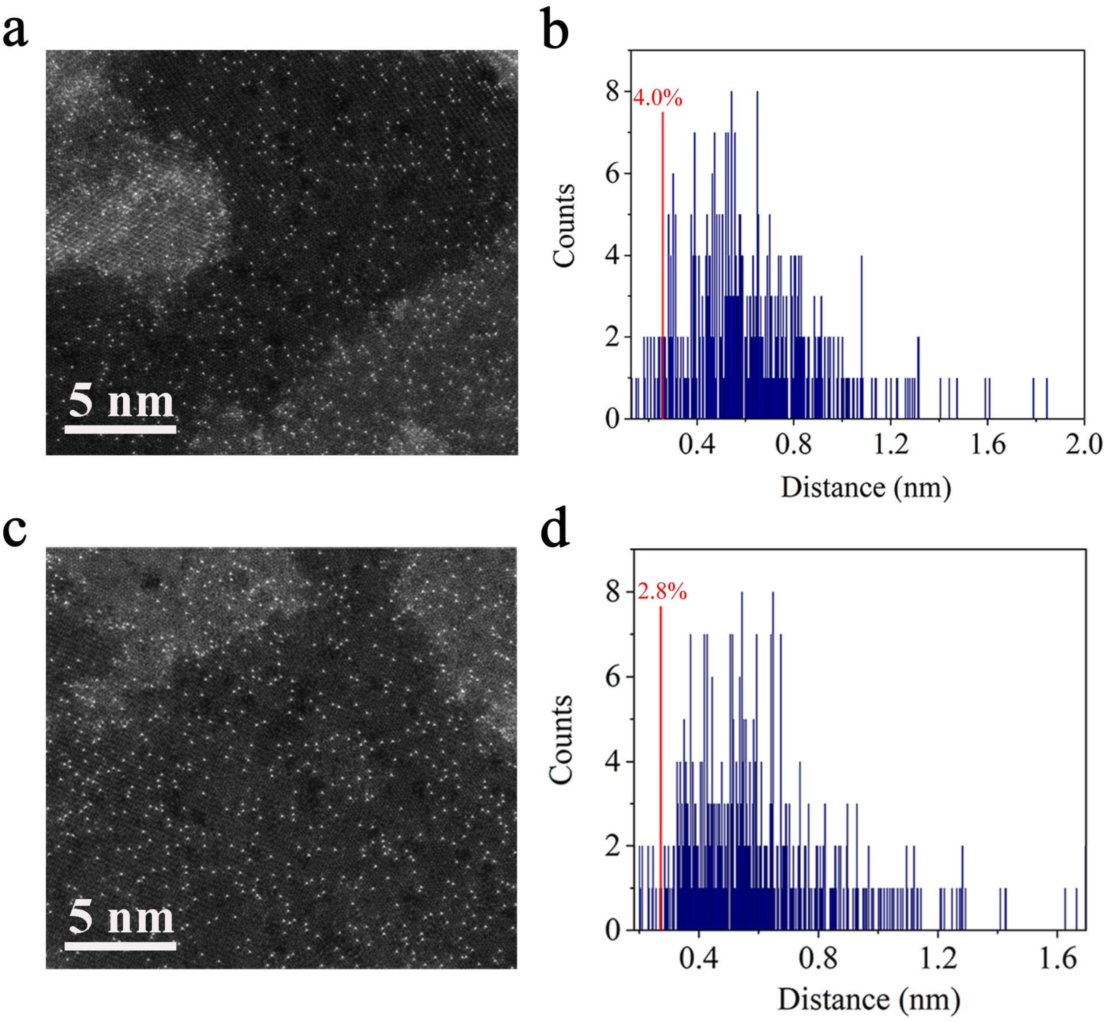

Supplementary Fig. 7. The distance between Cu single atoms in Cu-SA/$Ti_3C_2T_x$. (a, c) Atomic-resolution HAADF-STEM images of Cu-SA/$Ti_3C_2T_x$. (b, d) the corresponding distance distributions of Cu atoms (the bright spots). It was measured by the two nearest bright spots from *ca*. 450 Cu single atoms. An average of Cu–Cu interatomic distance of 0.61 nm was observed, larger than that of Cu–Cu bond (*ca*. 0.27 nm, see details in Supplementary Fig. 21) estimating from the optimized Cu dimer model.

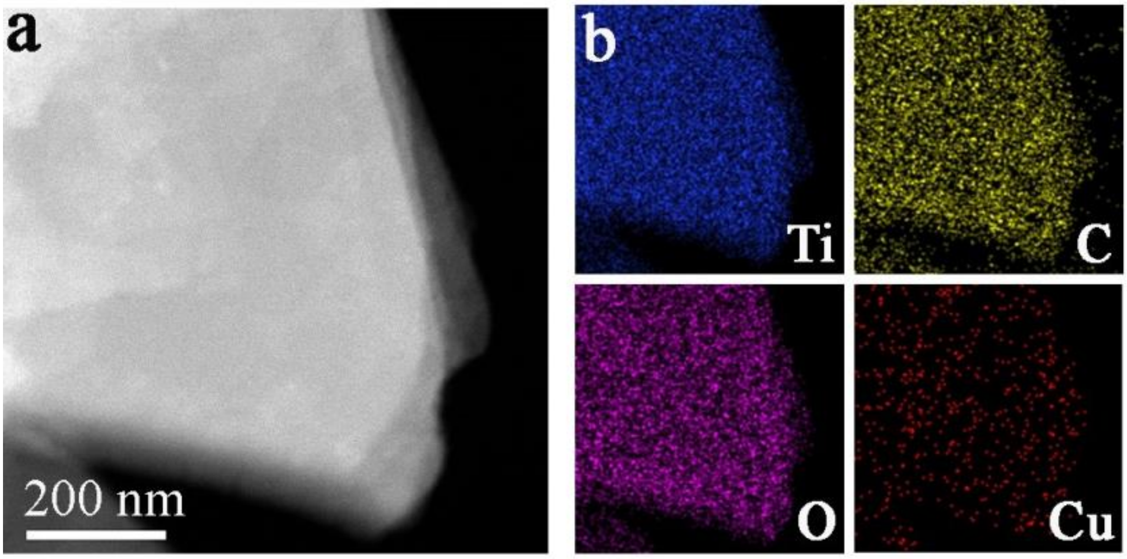

Supplementary Fig. 8. The EDX elemental mappings of Cu-SA/$Ti_3C_2T_x$. (a) HAADF-STEM image. (b) The corresponding EDX elemental mapping images of Cu-SA/$Ti_3C_2T_x$. Homogenous distribution of Ti, C, O and Cu was indicated throughout the surface in Cu-SA/$Ti_3C_2T_x$.

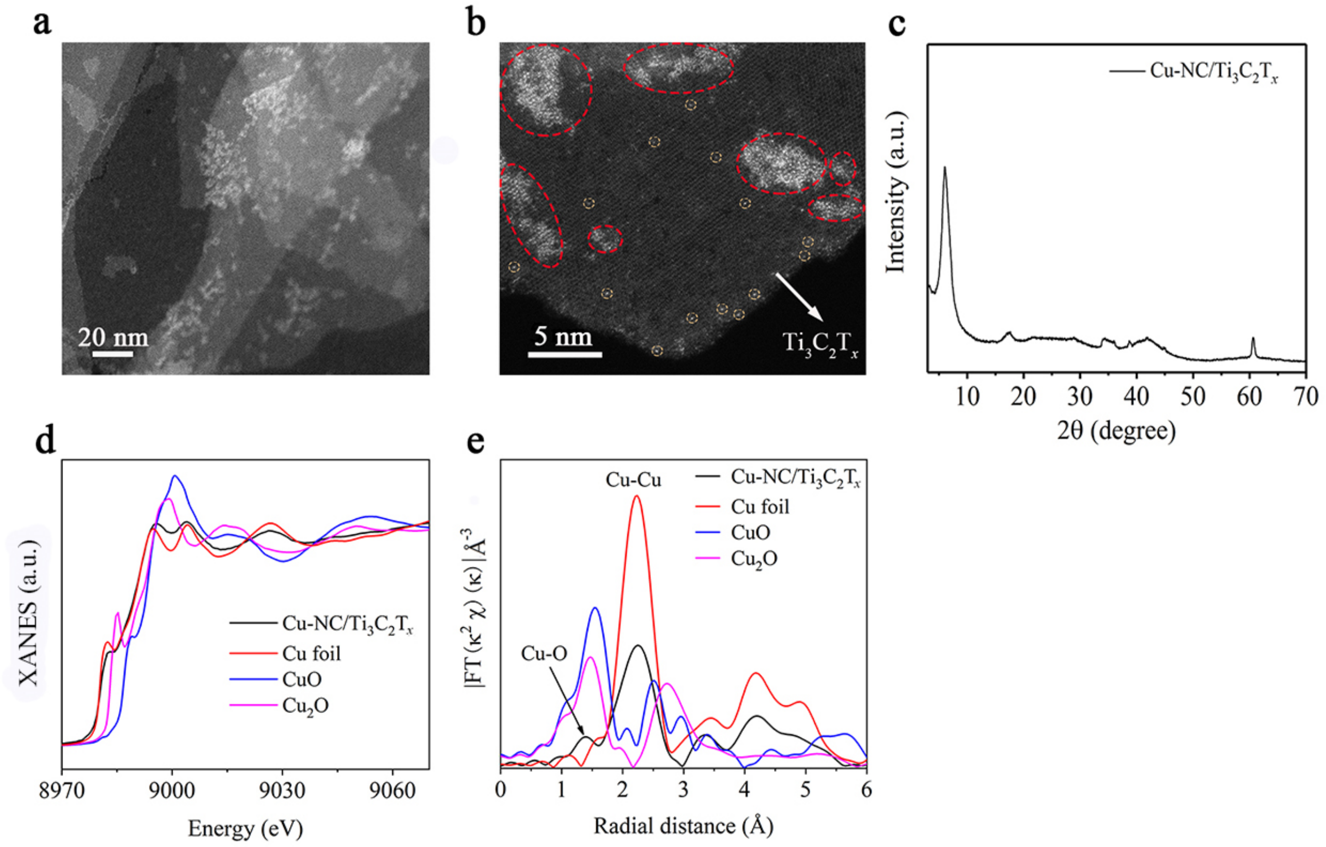

Supplementary Fig. 9. The structure characterizations of Cu-NC/$Ti_3C_2T_x$. (a) HAADF-STEM image, (b) Atomic-resolution HAADF-STEM image, (c) XRD pattern, (d) XANES spectra, and (e) FT-EXAFS curves at the Cu K-edge with CuO, Cu_2O and Cu foil as reference. Cu nanoclusters (red circles) and Cu single atoms (yellow circles) can be seen simultaneously on $Ti_3C_2T_x$ substrate in **b**. Furthermore, main peaks, associated with Cu–O and Cu–Cu scattering paths, were observed in the FT-EXAFS curve of Cu-NC/$Ti_3C_2T_x$.

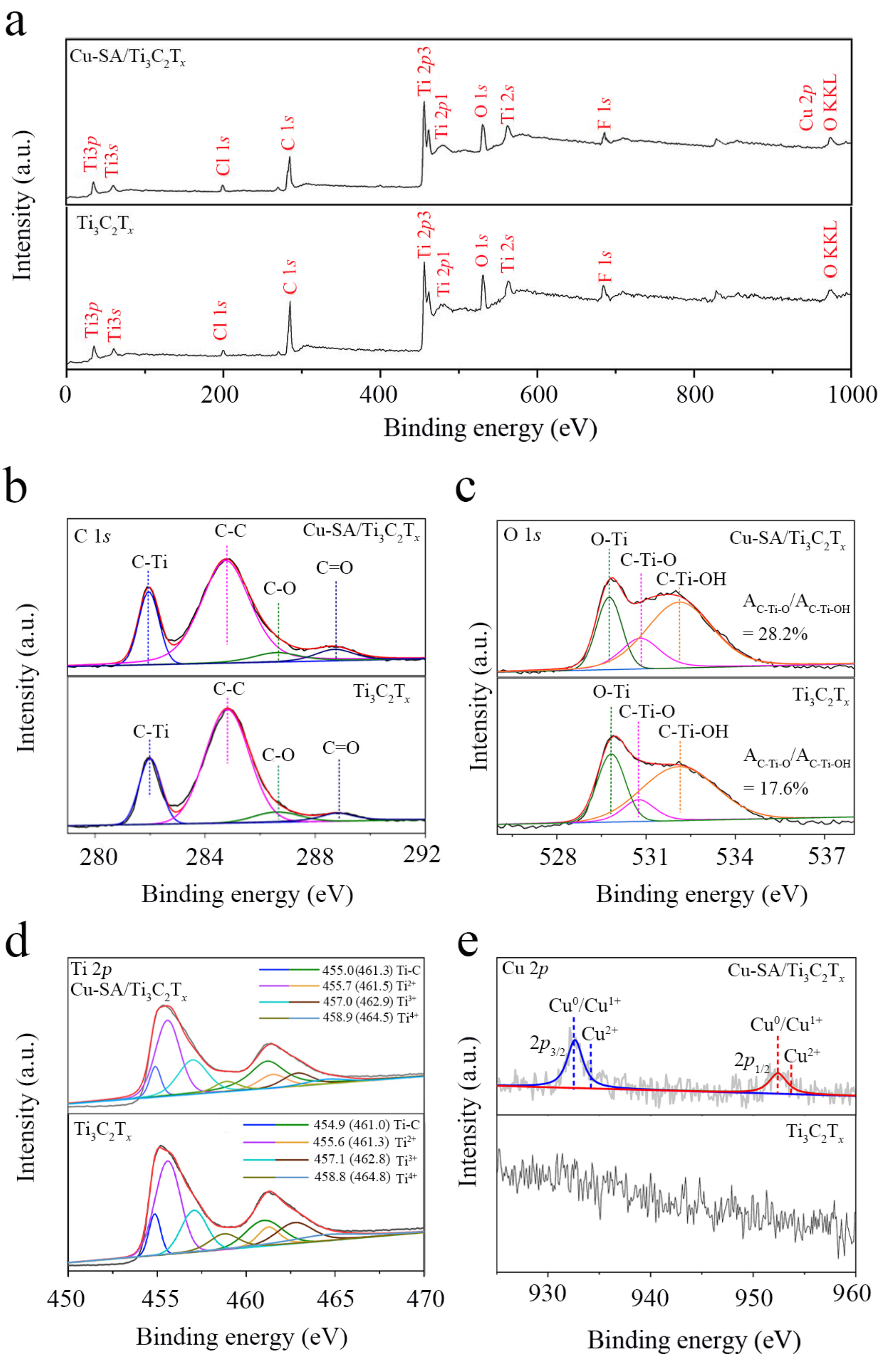

Supplementary Fig. 10. XPS results of Cu-SA/$Ti_3C_2T_x$ and pure $Ti_3C_2T_x$. (a) XPS survey, (b) C 1*s* XPS spectra, (c) O 1*s* XPS spectra, (d) Ti 2*p* XPS spectra, and (e) Cu 2*p* XPS spectra.

The peaks that correspond to C−Ti, O−Ti, C-Ti−O/OH, C−C and C-O/C=O bonds were deconvoluted[1, 2]. Note that the binding energy of Cu^0 and Cu^{1+} is too close to distinguish. Cu 2*p* peaks appeared at 932.5 and 952.4 eV for Cu-SA/$Ti_3C_2T_x$, suggesting the successful incorporation of Cu atoms. High-resolution Ti 2*p* XPS spectra show two peaks at around 455.5 and 461.3 eV, corresponding to Ti interactions with carbons and terminal atoms (i.e. C-Ti-T_x). The Ti^{4+} at 458.9 eV corresponds to Ti-O bonds on terminals. No obvious increase in Ti^{4+} state can be observed, indicating the well-preserved of Ti state in Cu-SA/$Ti_3C_2T_x$. The high-resolution C 1*s* XPS spectra show two peaks at 281.9 and 284.7 eV, corresponding to the C−Ti and C−C bonds. After the introduction of Cu atoms, no shift of C−C peak can be noticed, indicating no obvious interaction existed between Cu and C atoms. For high-resolution O 1*s* XPS spectra, the Ti−O, C−Ti−O and C−Ti−OH bonds can be fitted at 529.7, 530.8 and 532.0 eV, respectively. The ratio of C−Ti−O to C−Ti−OH bond increased from 17.6% to 28.2% after the introduction of Cu, agree with the formation of Cu single atoms in $Ti_3C_2T_x$.

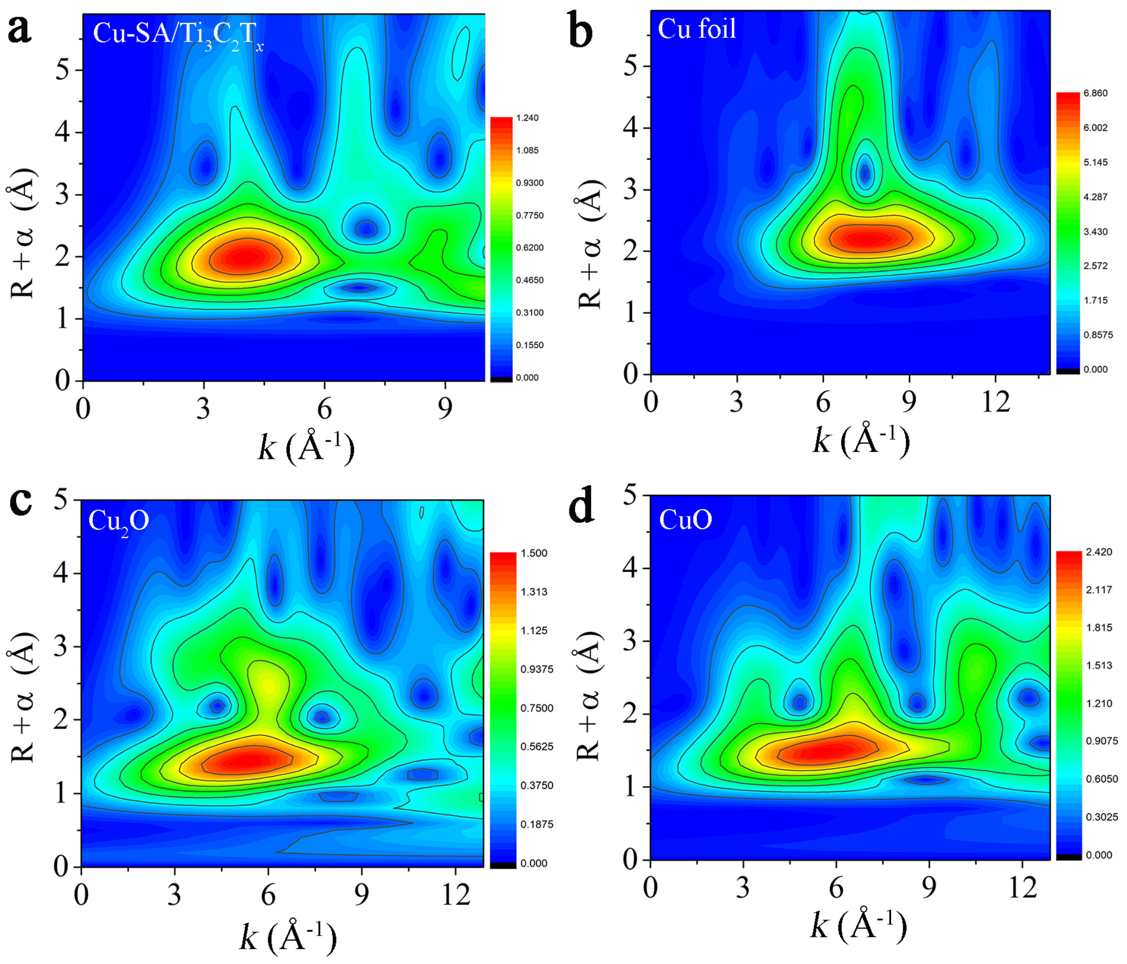

Supplementary Fig. 11. Wavelet transform plots the Cu K-edge EXAFS signals. (a) Cu-SA/$Ti_3C_2T_x$, (b) Cu foil, (c) Cu_2O, and (d) CuO. No intensity maximum was detected near 7.7 $Å^{-1}$ (Cu−Cu) for Cu-SA/$Ti_3C_2T_x$, further confirming that Cu single atoms are atomically dispersed.

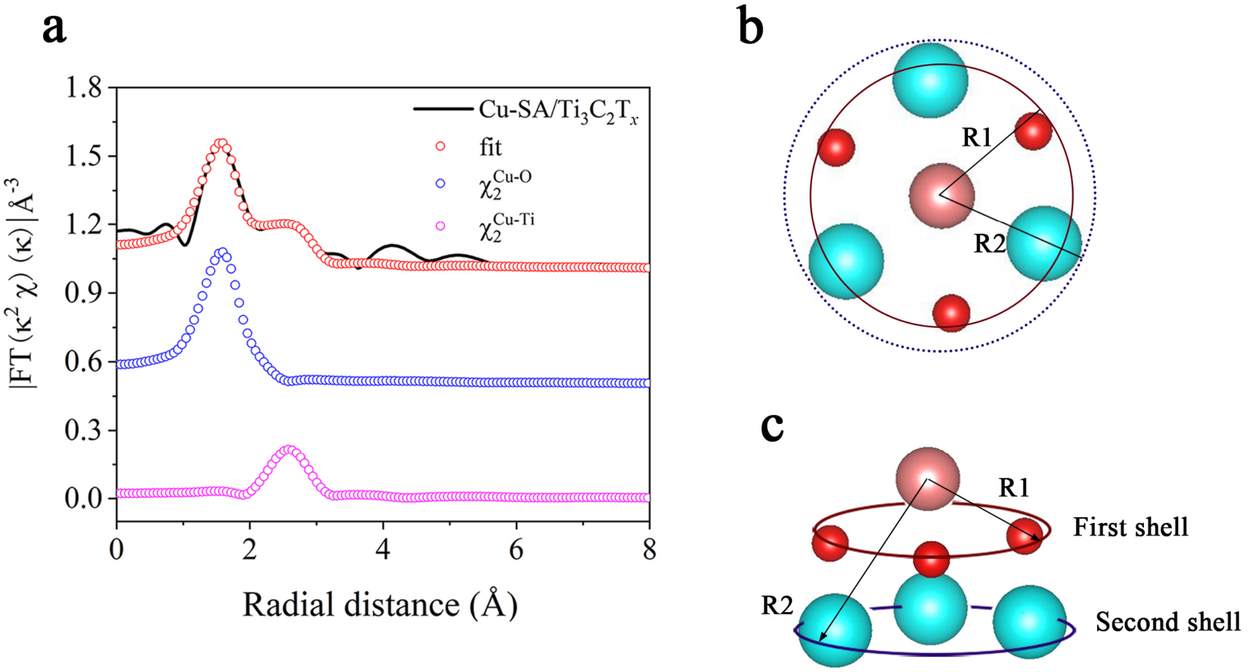

Supplementary Fig. 12. The fittings results of the FT-EXAFS spectrum for Cu-SA/$Ti_3C_2T_x$ and the schemes. (a) The first two-shell (O, Ti) fittings. (b, c) Schemes of coordination shells around Cu single atoms. The corresponding fitting parameters were listed in Supplementary Table 1.

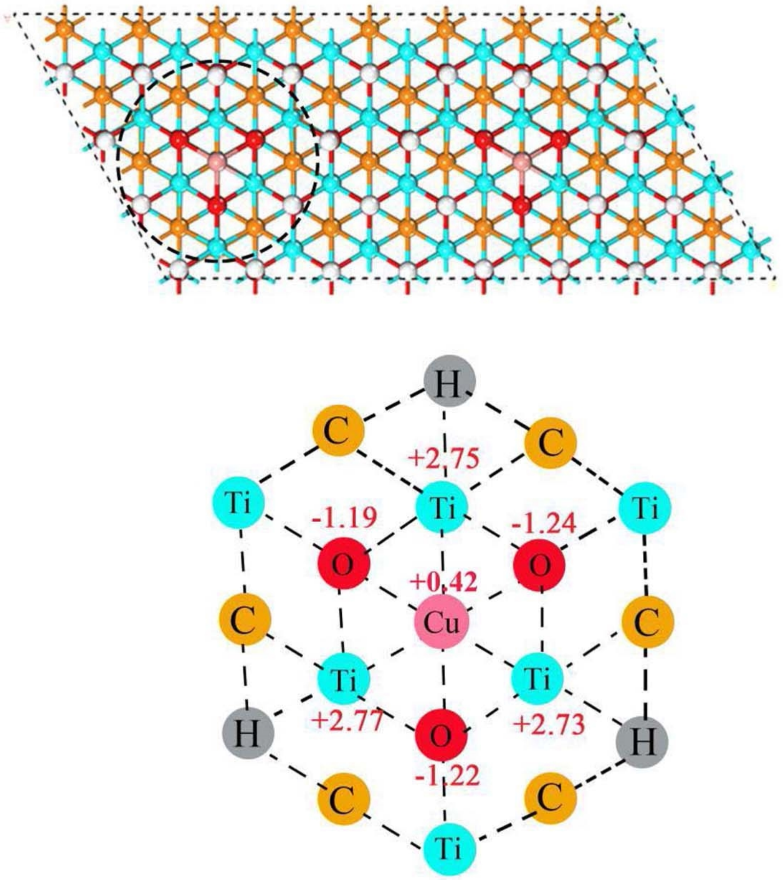

Supplementary Fig. 13. Bader charge in Cu-SA/$Ti_3C_2T_x$ model. Cu single atoms in Cu-SA/$Ti_3C_2T_x$ are positively charged (+0.42), in line with XANES result (Fig. 1e).

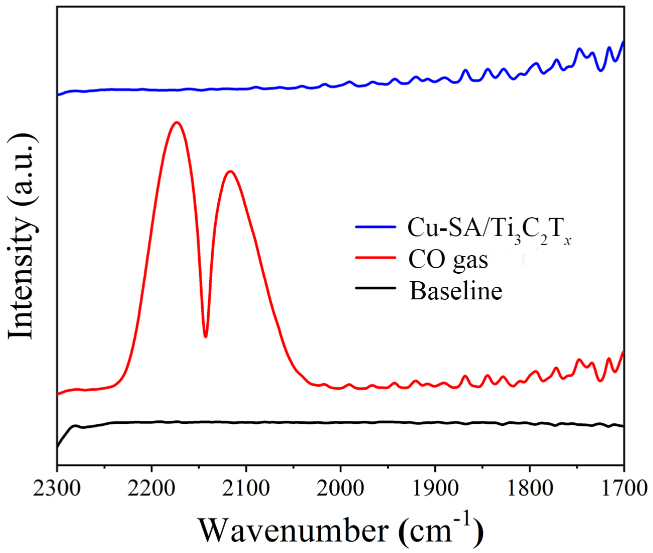

Supplementary Fig. 14. CO-DRIFTS spectra of Cu-SA/$Ti_3C_2T_x$. There is no characteristic peak corresponds to linearly bonded CO on Cu single atoms in Cu-SA/$Ti_3C_2T_x$ (blue curve). This finding may be ascribed to the weakening of CO adsorption on Cu single atom catalysts without an applied bias voltage.

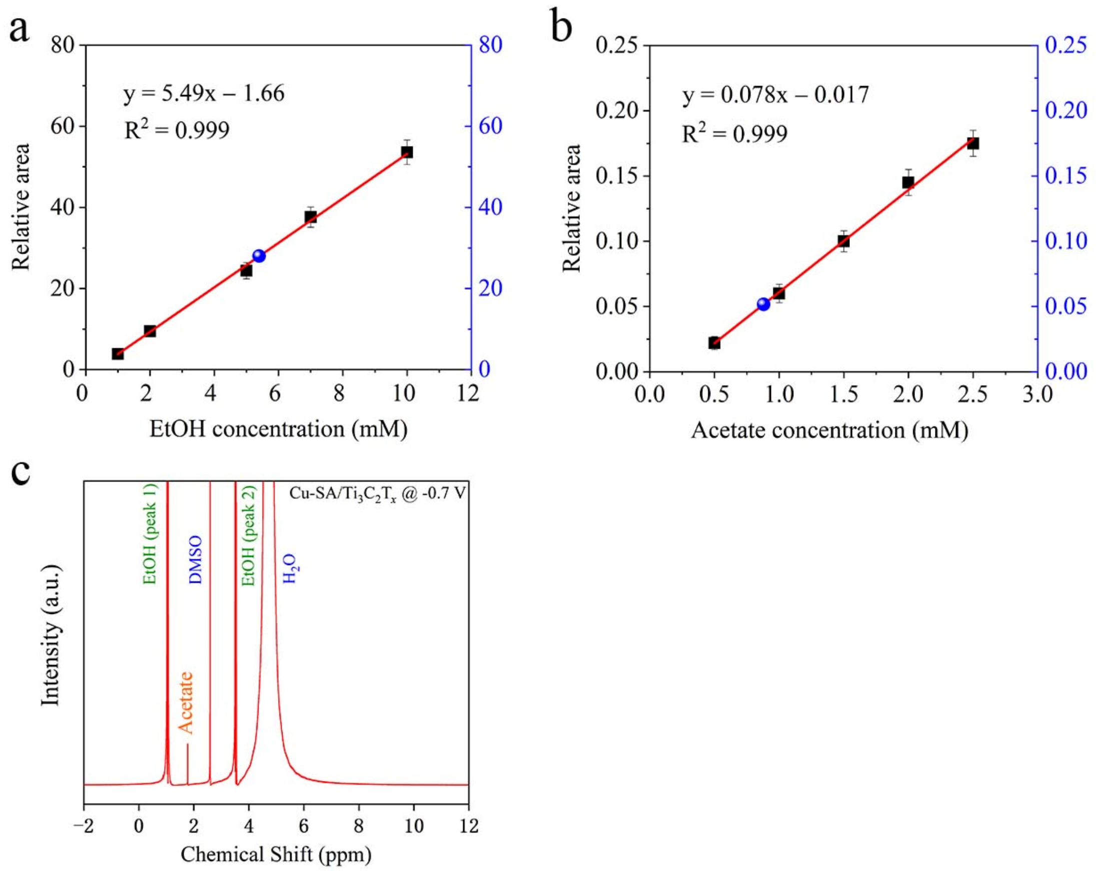

Supplementary Fig. 15. The standard curves and the representative ^{1}H NMR spectra of the liquid products. (a-b) Standard curves for EtOH (a) and acetate (b) products. (c) ^{1}H NMR spectra of the electrolyte taken after 2-h electrocatalysis over Cu-SA/$Ti_3C_2T_x$ at –0.7 V vs RHE. The standard curves were made using standard chemicals over the concentration range of interest (EtOH and acetate), with the internal standard DMSO in 1 M KOH. The linearity of the two curves is as high as 0.999. After the 2-h electrolysis at −0.7 V vs RHE, the ratios of the areas of the produced acetate and EtOH (peak 1) to the DMSO peak area were calculated to be 27.98 and 0.05164, respectively. The obtained ratios were then compared to the standard curves (**a** and **b**) to quantify the concentrations of the reaction products. Accordingly, the concentrations of EtOH and acetate (the blue balls in **a** and **b**) were measured to be 5.4 and 0.88 mM, respectively.

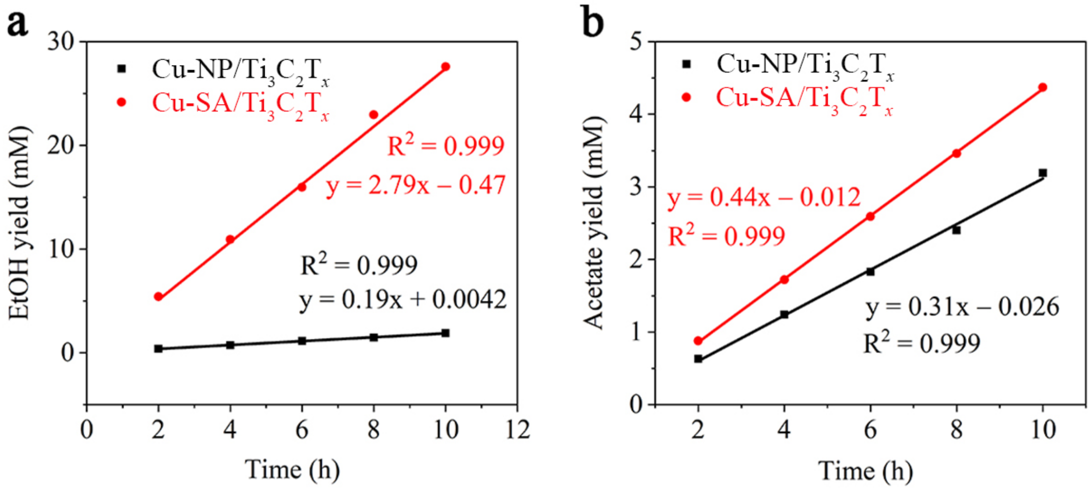

Supplementary Fig. 16. The yield of liquid products vs reaction time for of Cu-SA/$Ti_3C_2T_x$ and Cu-NP/$Ti_3C_2T_x$ at −0.7 V vs RHE. (a) EtOH, and (b) acetate. The values of the EtOH or acetate yield rate can be derived from the slopes of the curves made by plotting the EtOH or acetate concentrations vs reaction times. Thus, the formation rates of EtOH and acetate over the Cu-SA/$Ti_3C_2T_x$ are calculated to be 2.79 and 0.44 mM h^{-1} at −0.7 V vs RHE, respectively. Similarly, for Cu-NP/$Ti_3C_2T_x$, the formation rates of EtOH and acetate are 0.19 and 0.31 mM h^{-1}, respectively.

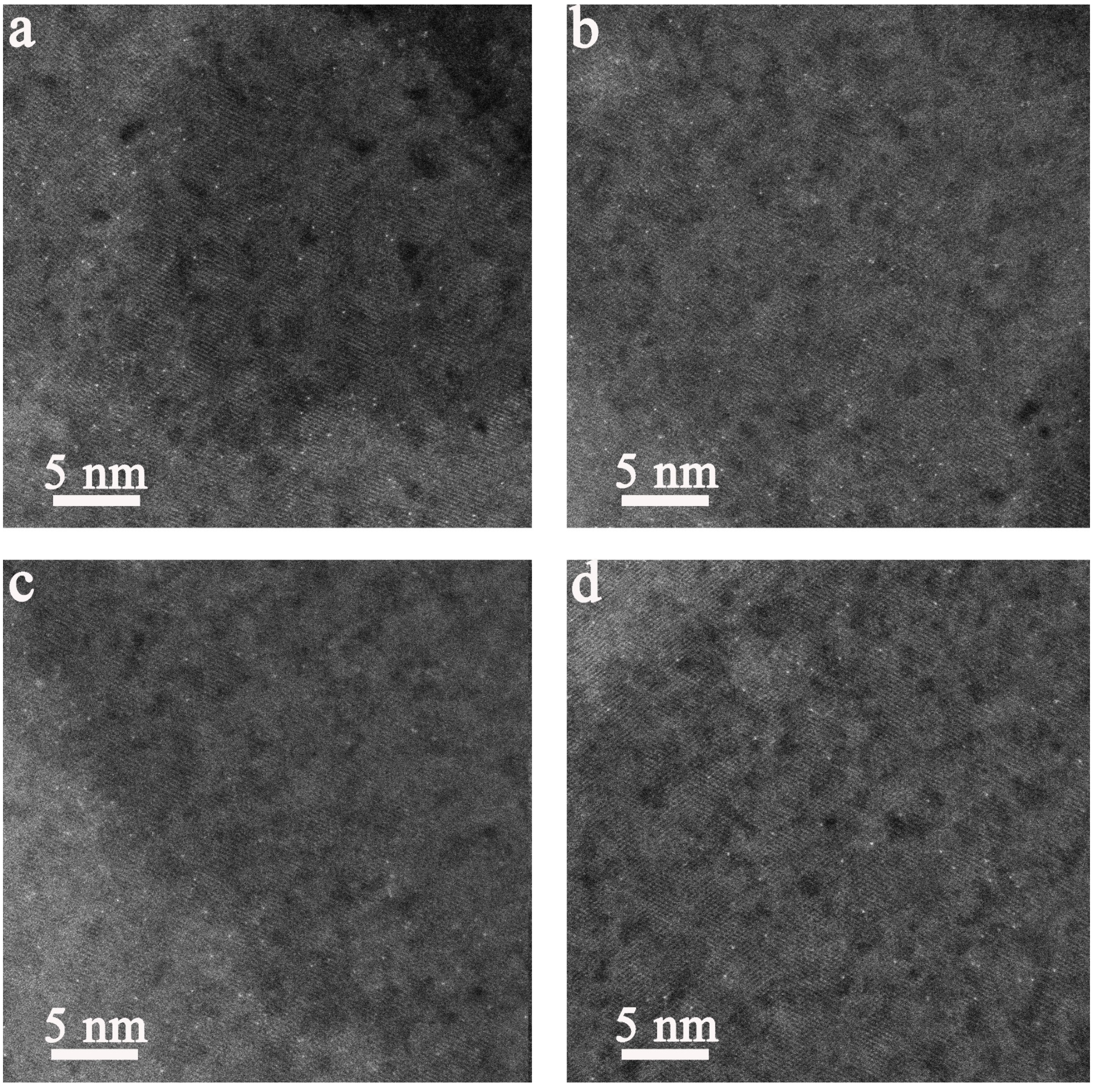

Supplementary Fig. 17. The atomic-resolution HAADF-STEM images of the Cu-SA/$Ti_3C_2T_x$ catalyst after stability test. The Cu single atoms were clearly observed and no Cu nanoparticles/clusters were formed, demonstrating the excellent stability of Cu single atoms.

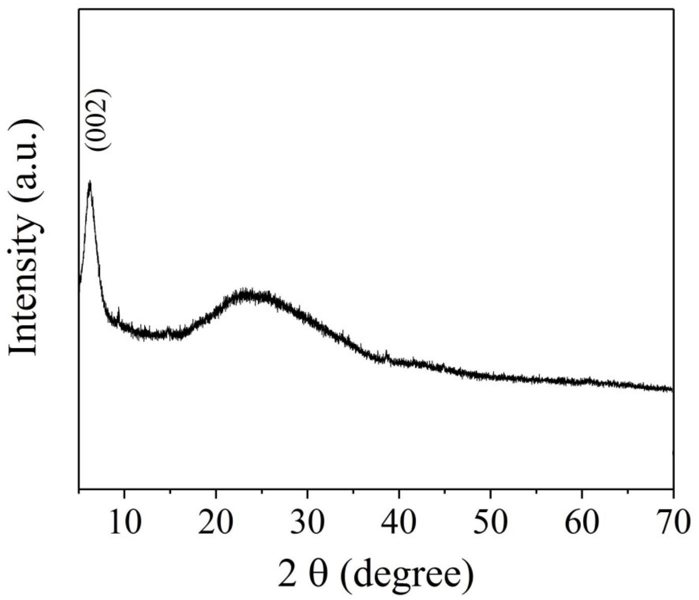

Supplementary Fig. 18. XRD pattern of Cu-SA/$Ti_3C_2T_x$ after stability test. No additional peaks appear compared with that of fresh Cu-SA/$Ti_3C_2T_x$ in Fig. 1b, demonstrating the good stability of Cu SAs. The weakening of (002) peak and the appearance of an amorphous broad peak around 24° were caused by the exfoliation of nanosheets resulting from the sonication process, as well as the intercalation of electrolyte ions during the electrolytic process[3]. The ^{13}CO isotopic labeling experiment confirmed that the carbon source in the gas and liquid products originated from the reduction of supplied gaseous CO (see details in Supplementary Figs. 22-24). Moreover, the carbon content of the fresh Cu-SA/$Ti_3C_2T_x$ and the used one is measured to be 35.7 and 36.2 wt%, respectively, indicative of the good stability of Cu-SA/$Ti_3C_2T_x$ during the CO reduction test.

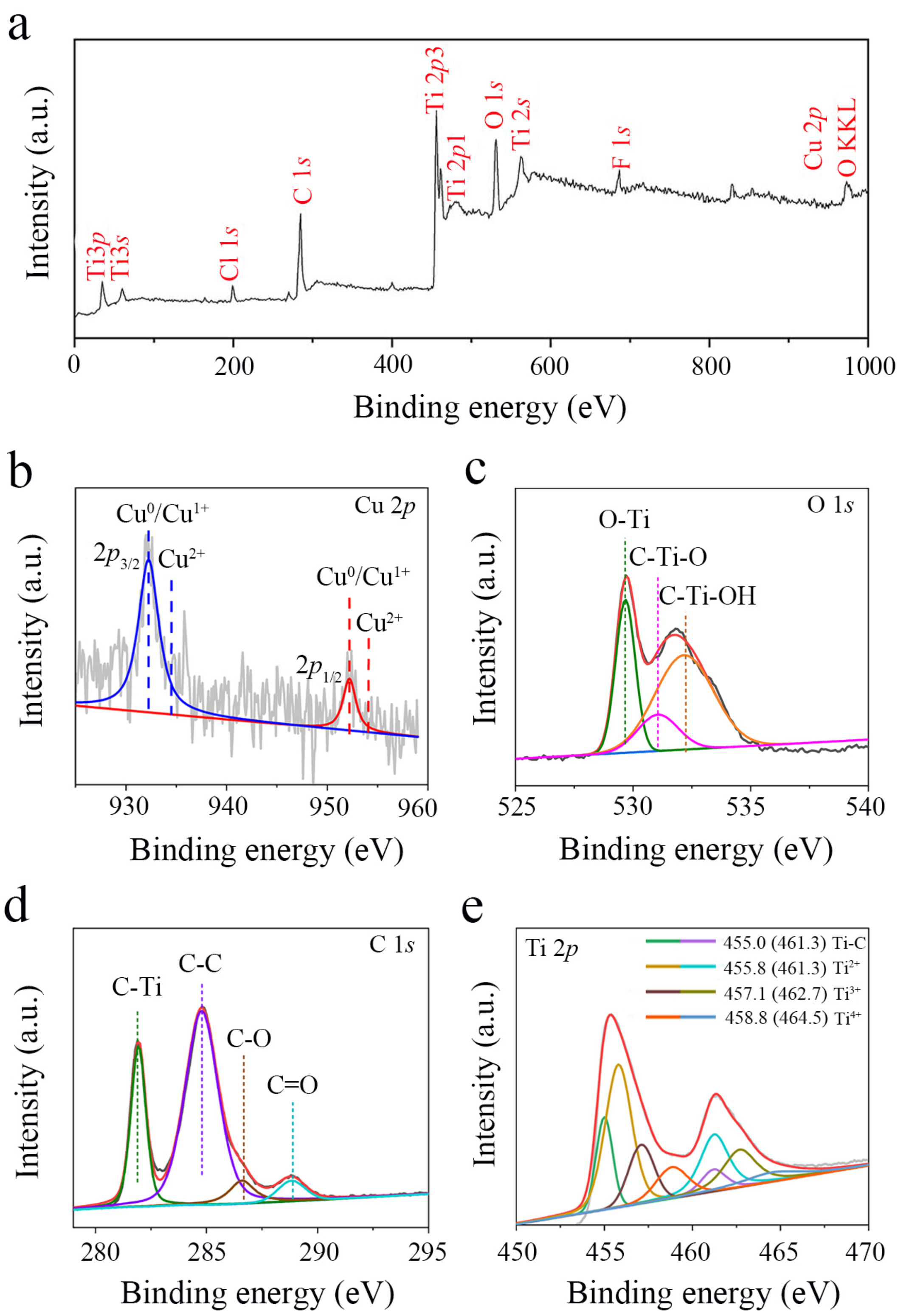

Supplementary Fig. 19. High-magnification XPS spectra of Cu 2*p* for Cu-SA/$Ti_3C_2T_x$ after stability test. (a) XPS survey, (b) Cu 2*p* XPS spectrum, (c) O 1*s* XPS spectrum, (d) C 1*s* XPS spectrum, and (e) Ti 2*p* XPS spectrum. No obvious change can be observed compared with that of fresh Cu-SA/$Ti_3C_2T_x$ (Supplementary Fig. 10), indicating the chemical stability of the Cu SAs.

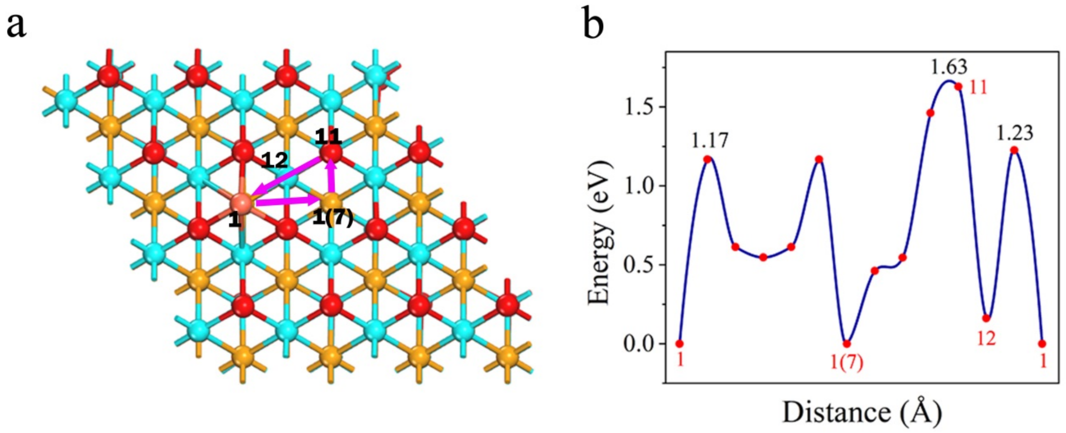

Supplementary Fig. 20. The migration of a single Cu atom on $Ti_3C_2T_x$ surface. (a) The diagram for the migration of a single Cu atom. (b) The corresponding diffusion energy barrier profiles. During the calculation, the O-terminated Cu-SA/$Ti_3C_2T_x$ model was used to calculate the migration process, considering that the Cu single atoms on O-terminated $Ti_3C_2T_x$ are easier to migrate than the OH-terminated ones. When the hydroxylated model was used, unexpected energy change caused by the H-escaping on $Ti_3C_2T_x$ surface would be introduced, which makes the total free energy fluctuated. To estimate the stability of Cu SA on $Ti_3C_2T_x$, the binding energy of Cu-SA/$Ti_3C_2T_x$ ($E^{\text{bind}}_{\text{Cu-SA/Ti}_3\text{C}_2\text{T}_x}$) was calculated. This binding energy is found to be +2.16 eV, smaller than the cohesive energy of +3.48 eV for bulk Cu ($E^{\text{coh}}_{\text{Cu}_{\text{bulk}}}$); however, it should be noted that this binding strength is still quite strong and agrees well with previous reports for SA catalysts[4, 5]. Besides, the kinetic stability that affecting the stability in practical electrocatalysis should be taken into consideration as well. Thus, to estimate the kinetic stability of Cu-SA/$Ti_3C_2T_x$, the surface mobility of Cu SAs was discussed by simulating the mobilization of a Cu SAs on $Ti_3C_2T_x$ surface using the CI-NEB approach. All the possible migration paths were considered. It shows that the energy barrier for the mobilization of a Cu SA from this most stable trap (1) to an adjacent triangle oxygen trap (1(7)) or Ti top site (12) is 1.17 eV and 1.23 eV, respectively. Another migration pathway is also considered from the (1(7)) site to an adjacent oxygen top site (1(7) → 11), which yields a greater energy barrier of 1.63 eV. The energy barrier is comparable to those reported Pd SAs (1.67 eV)[6] and Cu SAs (0.95 eV)[4] on CeO_2 (111), which indicates that the Cu SA on the $Ti_3C_2T_x$ surface is difficult to move away from the triangle oxygen traps, suggesting the high kinetic stability of Cu-SA/$Ti_3C_2T_x$.

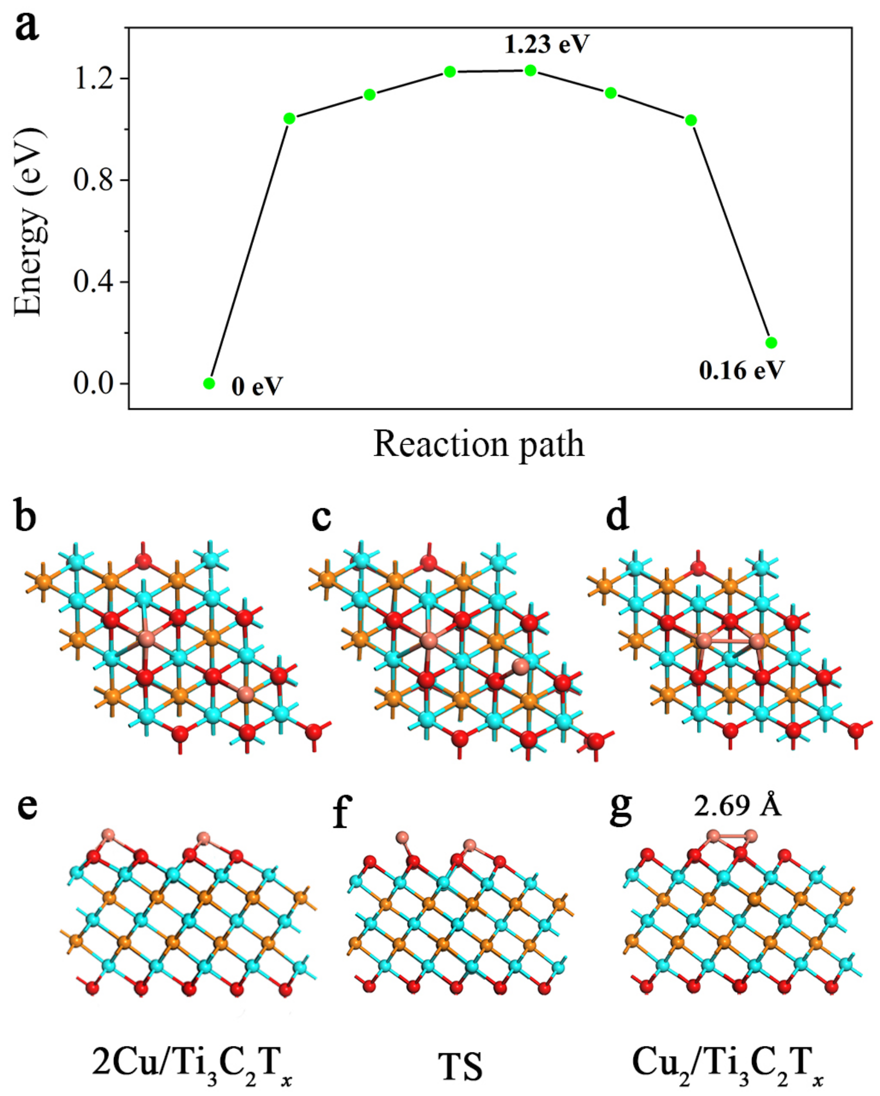

Supplementary Fig. 21. The dimerization process for two Cu atoms on $Ti_3C_2T_x$ Surface. (a) The energy profile. (b-g) The corresponding top (b, c, d) and side views (e, f, g) of the structures. TS stands for the transition state. During the calculation, O-terminated Cu-SA/$Ti_3C_2T_x$ model was used to calculate the aggregation process of Cu SAs. A possible instability for single Cu atoms is the appearance of Cu-dimer species. Therefore, the formation of Cu dimers was also investigated using CI-NEB method. It indicated that two adjacent Cu atoms need a relatively high energy barrier of 1.23 eV to form a Cu dimer through atom migration, indicating that Cu dimer is hard to be formed, consistent with theoretical calculations in Supplementary Fig. 20.

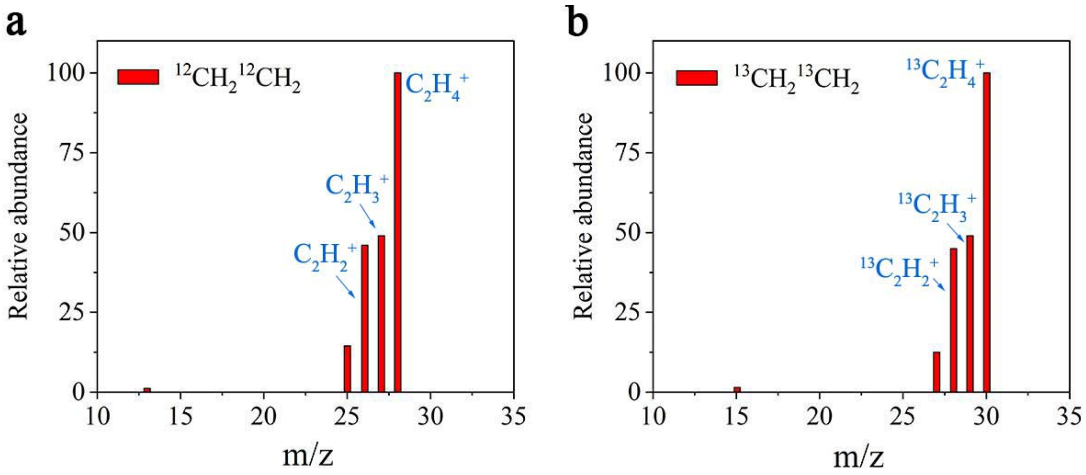

Supplementary Fig. 22. Typical mass spectra of the C_2H_4 for Cu-SA/$Ti_3C_2T_x$ reduction products. (a) Standard C_2H_4, (b) ^{13}C-C_2H_4 sample.

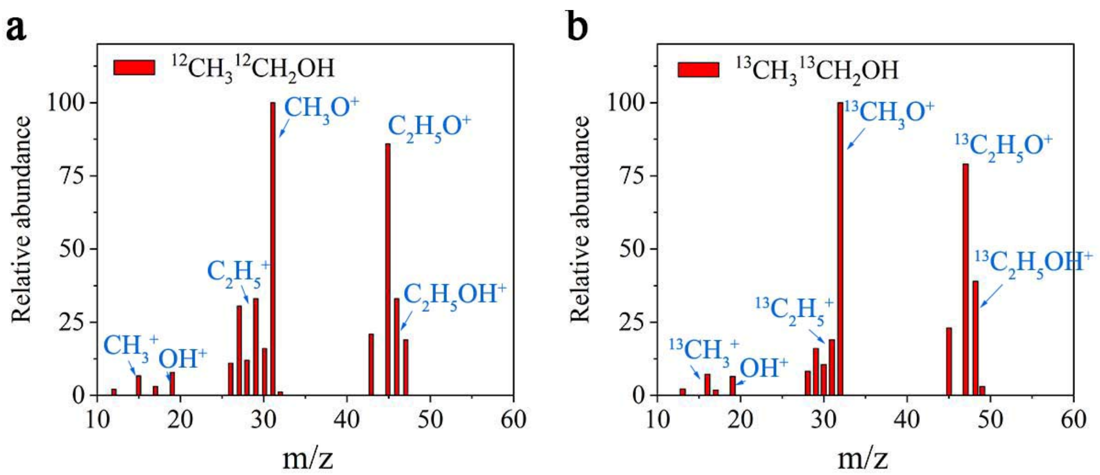

Supplementary Fig. 23. Typical mass spectra of EtOH for Cu-SA/$Ti_3C_2T_x$ reduction products. (a) Standard EtOH, (b) ^{13}C-EtOH sample. Note that the signal at m/z = 47 in **a** is due to the natural abundance of ^{13}C (refs.[7, 8]).

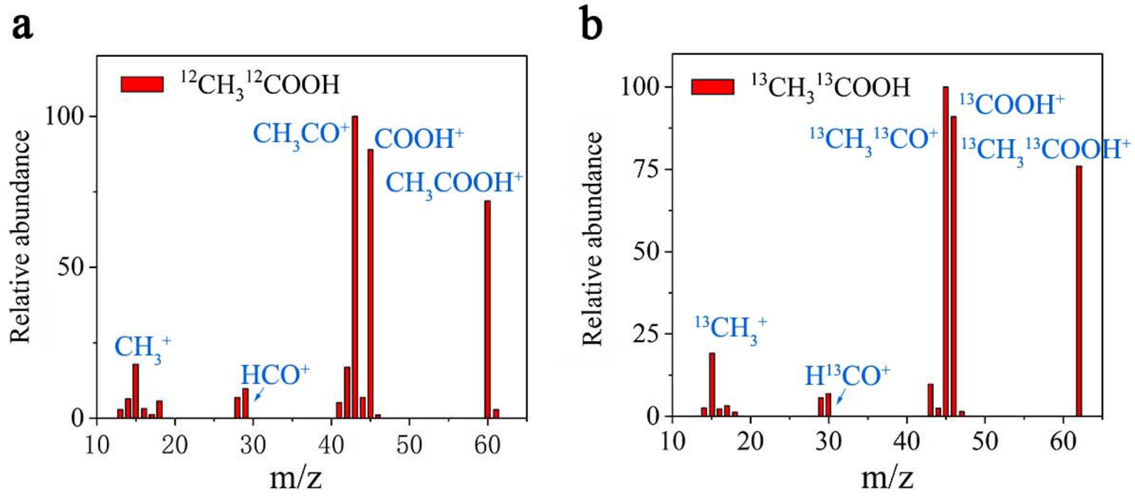

Supplementary Fig. 24. Typical mass spectra of the acetic acid for Cu-SA/$Ti_3C_2T_x$ reduction products. (a) Standard acetic acid, (b) ^{13}C acetic acid sample.

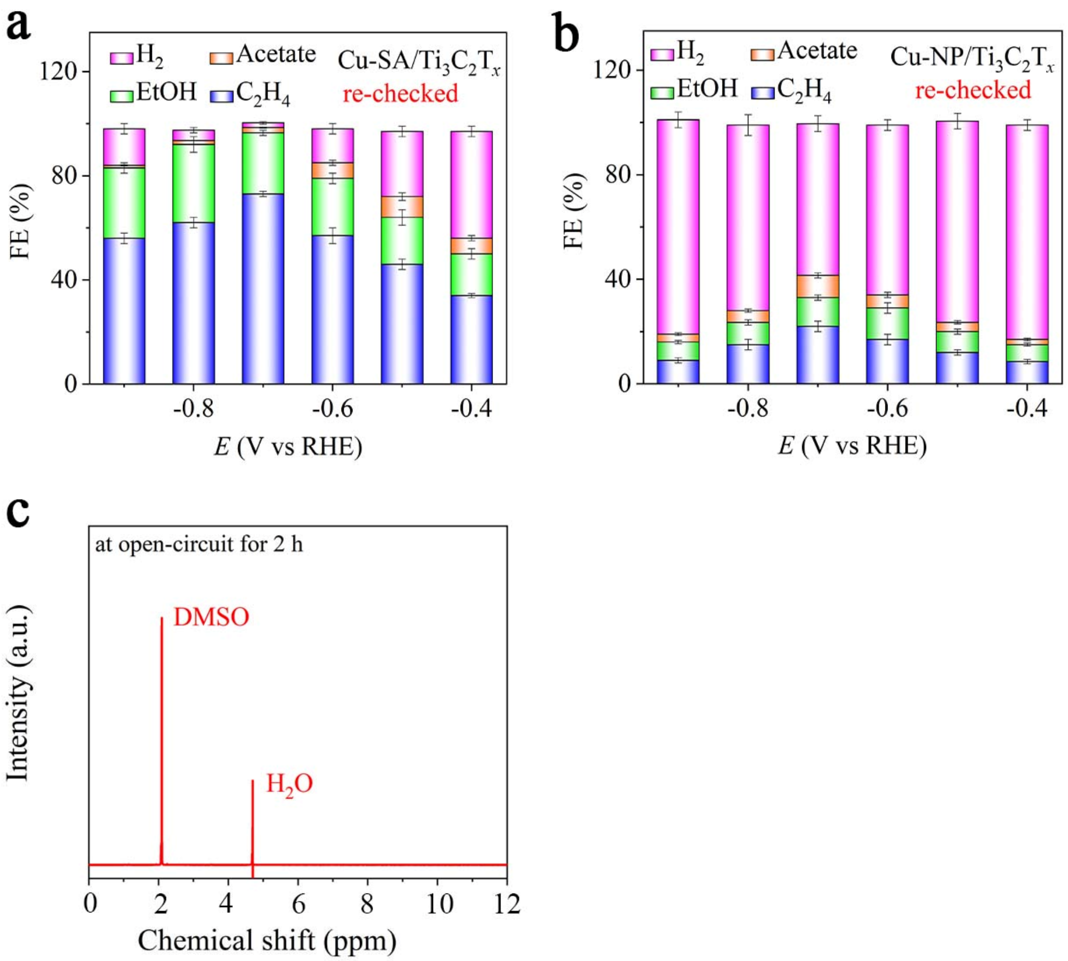

Supplementary Fig. 25. The rechecked Faradaic efficiencies and the control experiment. (a, b) The rechecked Faradaic efficiencies (FEs) toward CO reduction of Cu-SA/$Ti_3C_2T_x$ and Cu-NP/$Ti_3C_2T_x$ for 2 h at various applied potentials. In this control experiment, EtOH was replaced by isopropanol in the preparation of the ink. (c) The ^{1}H NMR analysis of liquid products when the electrolysis experiment was conducted under an open-circuit voltage. The error bars correspond to the standard deviations of measurements over three separately prepared samples under the same testing conditions. Cu-SA/$Ti_3C_2T_x$ and Cu-NP/$Ti_3C_2T_x$ achieved a FE for C_2 products of 98.5% and 41.5%, respectively, both of which are very close to those of FE values by using the ink containing EtOH. In addition, the maximum FE for EtOH production reaches 30% at −0.8 V vs RHE when the ink-containing isopropanol was employed−equal to the FE value by using the ink containing EtOH. Furthermore, when the electrolysis experiment was conducted under an open-circuit voltage, no EtOH can be detected after the electrolysis in **c**. These results suggest that the EtOH was generated from the electroreduction of dissolved CO by Cu-SA/$Ti_3C_2T_x$.

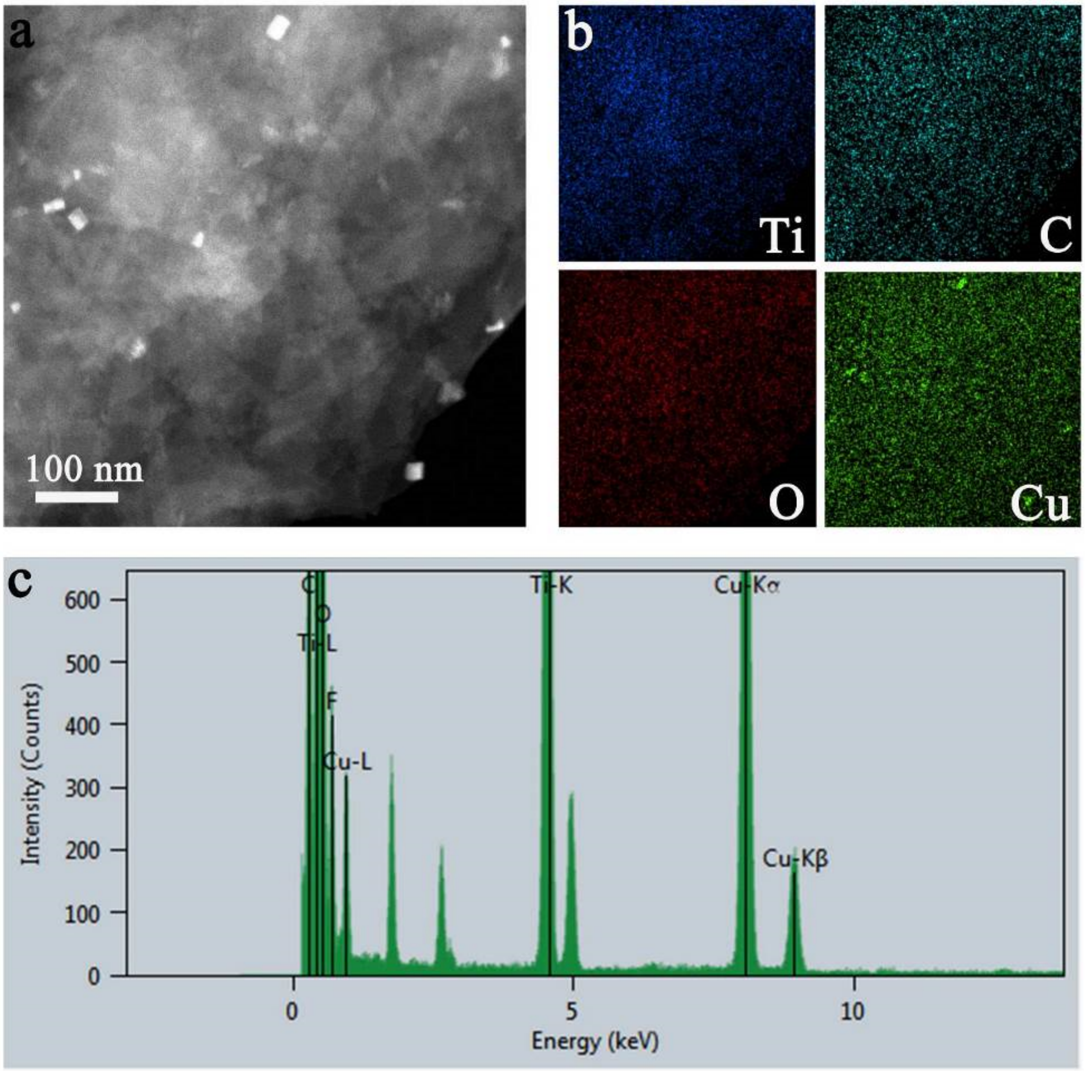

Supplementary Fig. 26. The EDX mappings of Cu-NP/$Ti_3C_2T_x$. (a) HAADF-STEM image. (b) EDX mapping images. (c) The EDX element composition analysis.

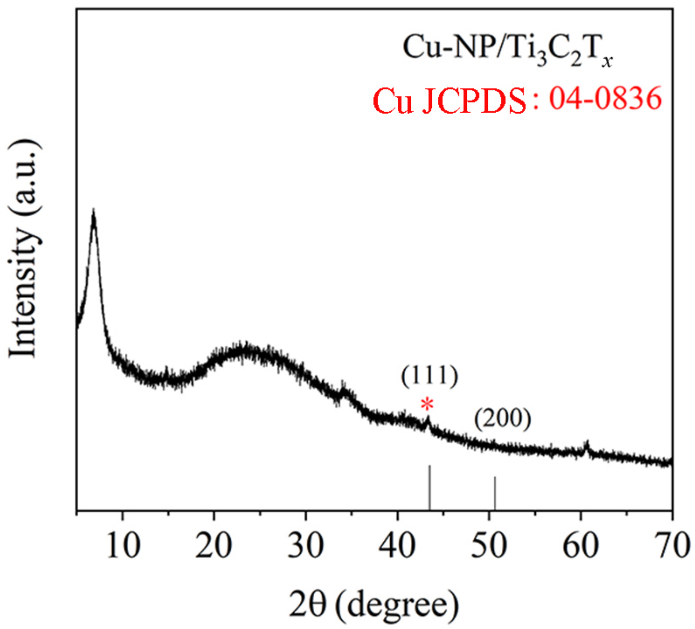

Supplementary Fig. 27. XRD pattern of Cu-NP/$Ti_3C_2T_x$. It showed a peak at ~43.3°, corresponding to the diffraction of (111) facets in Cu crystals.

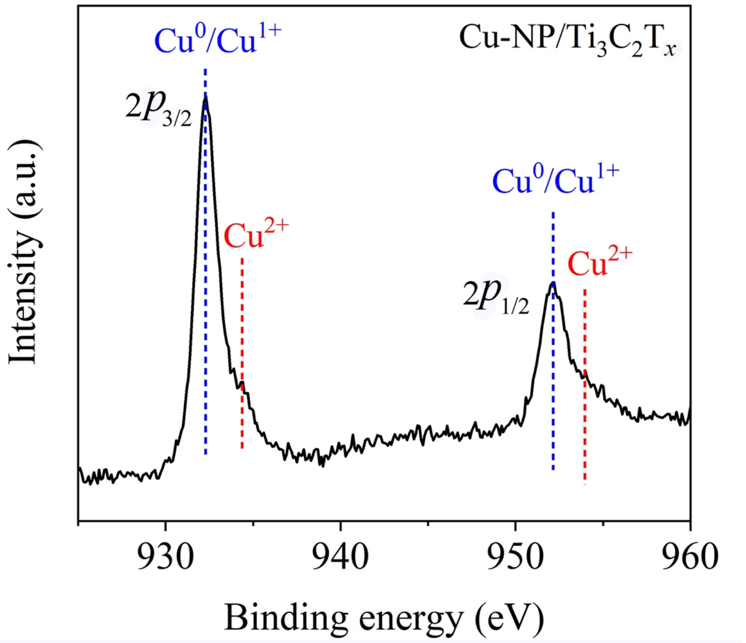

Supplementary Fig. 28. High-resolution Cu 2*p* XPS in the as-prepared Cu-NP/$Ti_3C_2T_x$ sample. It was found that two characteristic peaks located at 952.2 eV (Cu $2p_{1/2}$) and 932.3.0 eV (Cu $2p_{3/2}$). A small amount of Cu^{2+} can also be fitted, indicating a tinny amount of CuO formed. Note that the binding energy of Cu^0 and Cu^{1+} is too close to distinguish.

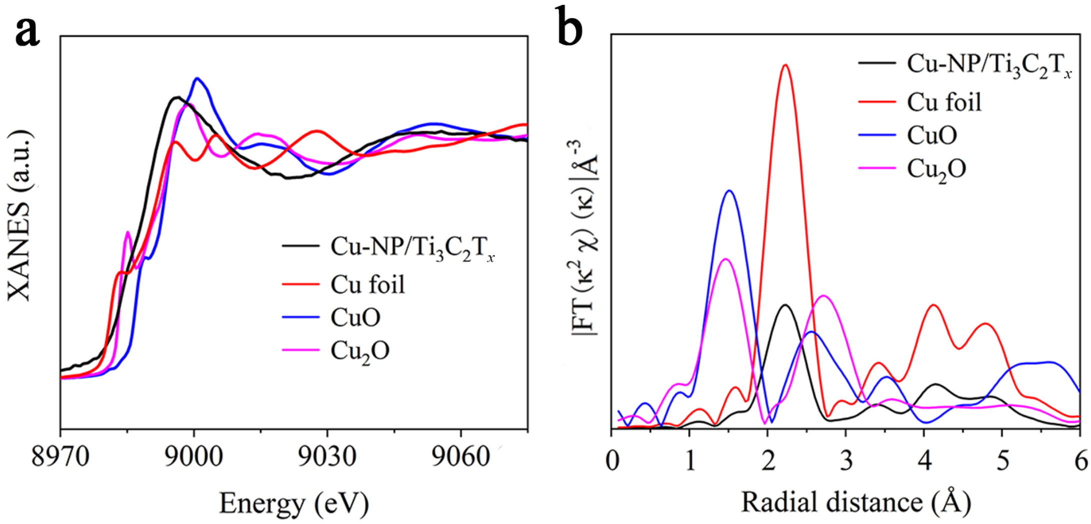

Supplementary Fig. 29. Structural characterization of Cu-NP/$Ti_3C_2T_x$. (a) XANES spectra at the Cu K-edge with CuO, Cu_2O and Cu foil as reference. (b) FT-EXAFS curves in which χ(k) denotes the EXAFS oscillation function. A reflection from the Cu–Cu contribution (2.2 Å) is observed for Cu-NP/$Ti_3C_2T_x$, confirming the formation of Cu nanoparticles.

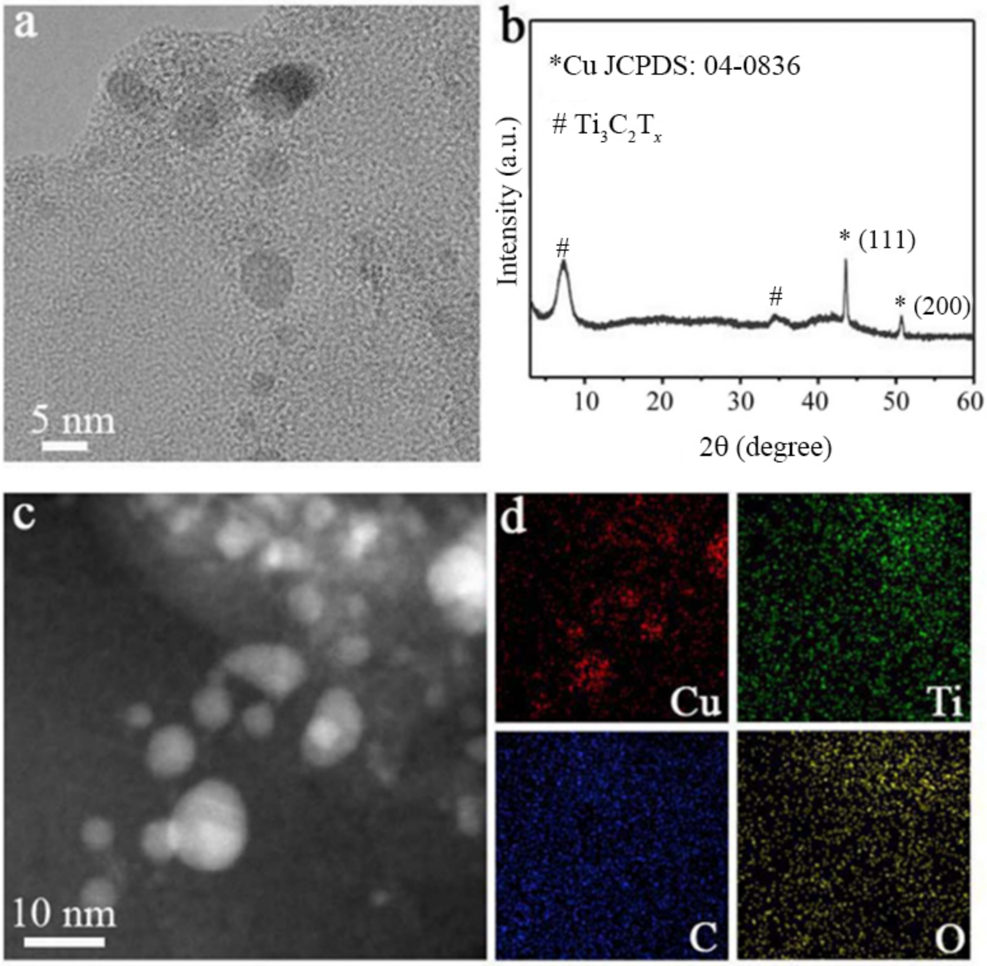

Supplementary Fig. 30. Characterization of Cu-NP/$Ti_3C_2T_x$-9.8. (a) TEM image, (b) XRD pattern, (c, d) HAADF-STEM image and the corresponding EDX mapping images of Cu, Ti, C, O elements. Note that 9.8 represents the actual Cu loading in wt%.

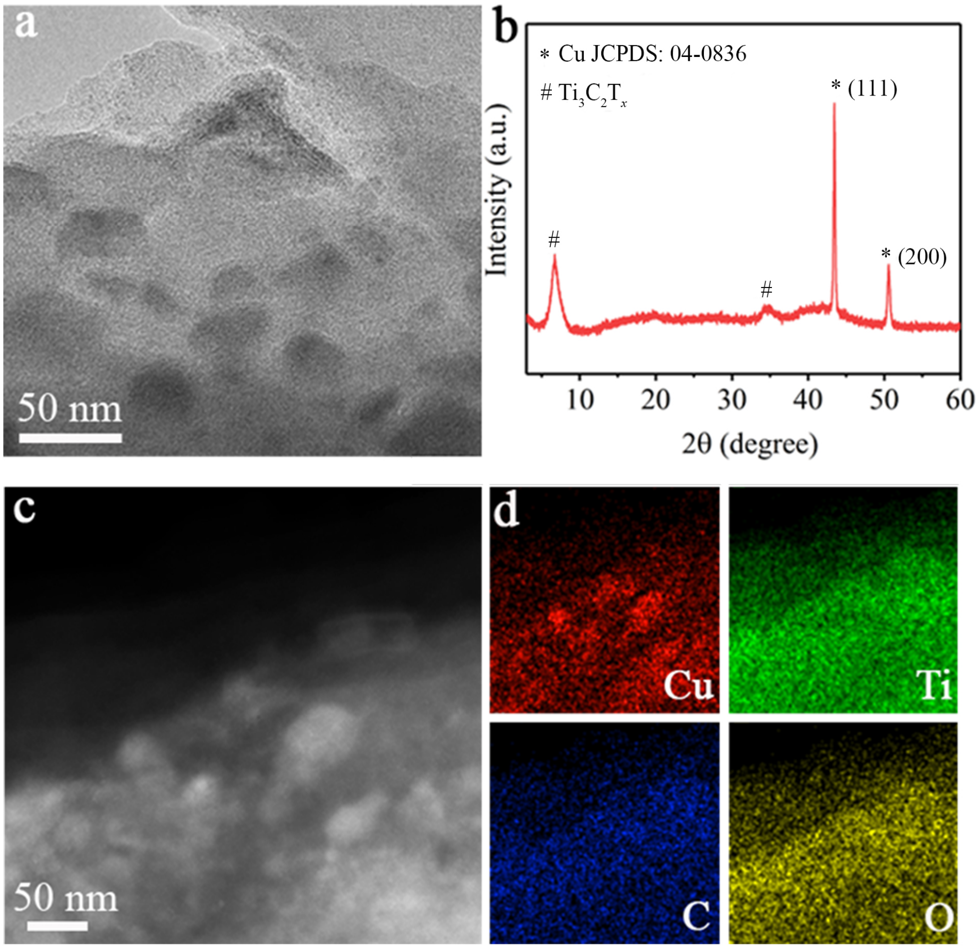

Supplementary Fig. 31. Characterization of Cu-NP/$Ti_3C_2T_x$-20.3. (a) TEM image, (b) XRD pattern, (c, d) HAADF-STEM image and the corresponding EDX mapping images of Cu, Ti, C, O elements. Note that 20.3 represents the actual Cu loading in wt%.

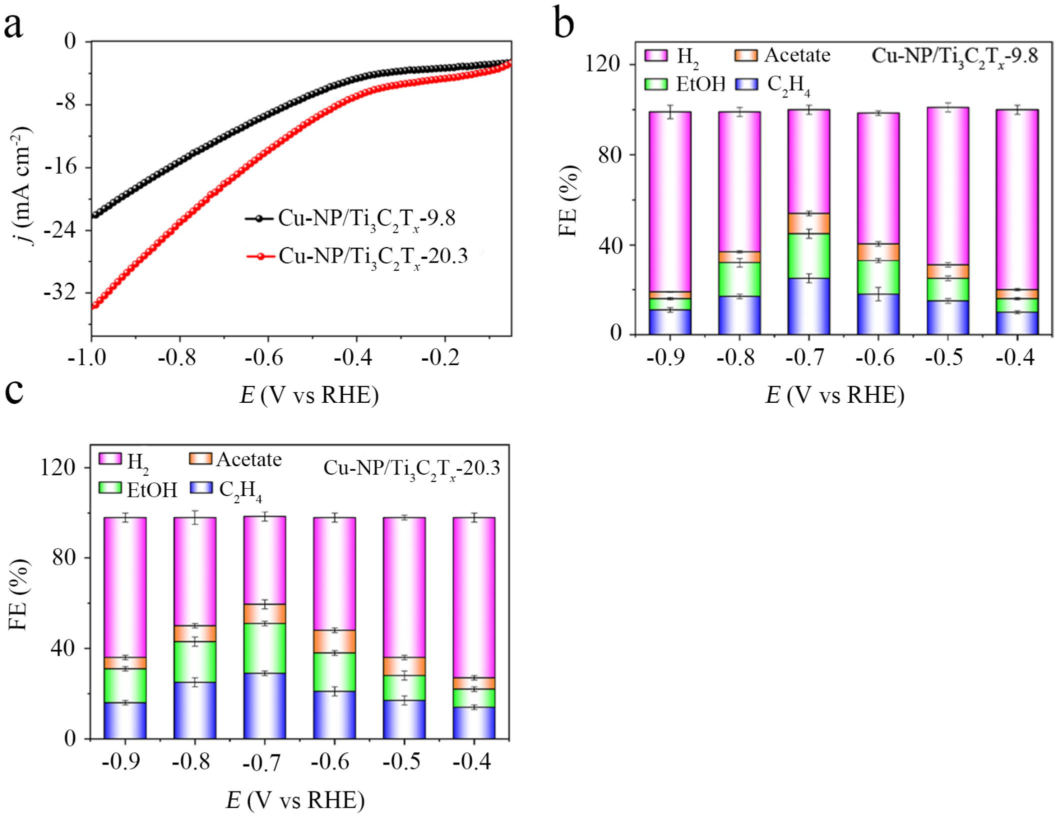

Supplementary Fig. 32. CO reduction performances of Cu-NP/$Ti_3C_2T_x$-9.8 and Cu-NP/$Ti_3C_2T_x$-20.3. (a) LSV curves measured in CO-saturated 1 M KOH solution. (b, c) Faradaic efficiencies (FEs) toward H_2 and COR products. The error bars correspond to the standard deviations of measurements over three separately prepared samples under the same testing conditions. The Cu-NP/$Ti_3C_2T_x$-9.8 and Cu-NP/$Ti_3C_2T_x$-20.3 afford improved reduction current densities of −21.97 and −34.0 mA cm^{-2} at −1.0 V vs RHE in comparison with that of Cu-NP/$Ti_3C_2T_x$ (−16.2 mA cm^{-2}; see Fig. 2a). Meantime, Cu-NP/$Ti_3C_2T_x$-9.8 and Cu-NP/$Ti_3C_2T_x$-20.3 exhibited the maximum FE of 54.0% and 59.5% at −0.7 V vs RHE for C_2 products, respectively, both of which are still inferior to those of Cu-SA/$Ti_3C_2T_x$ (Fig. 2b).

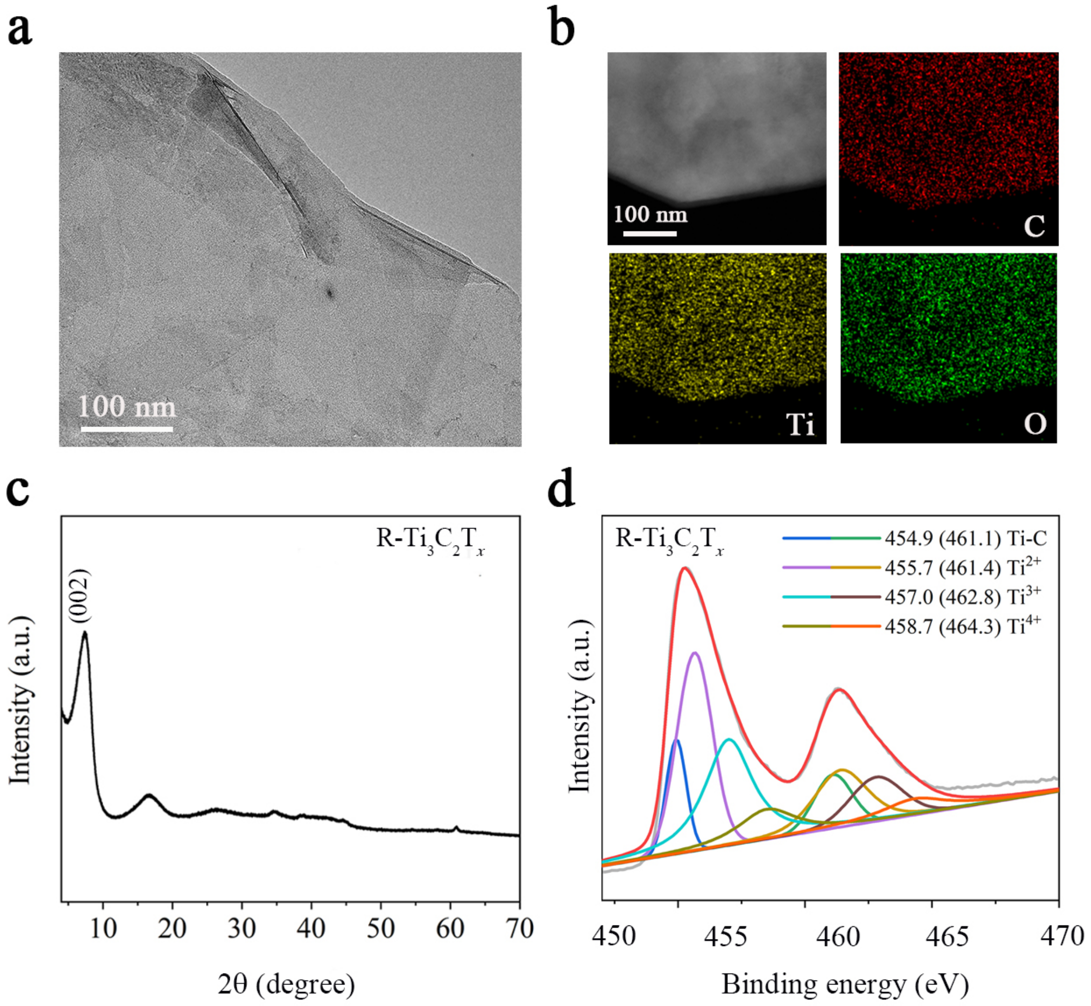

Supplementary Fig. 33. The structure characterization of R-$Ti_3C_2T_x$. (a) TEM image, (b) EDX elemental mapping images, (c) XRD pattern, and (d) Ti 2*p* XPS spectrum. It shows a nanosheet morphology with Ti, C and O homogenously-distributed. The XRD pattern exhibited the main peak representing the (002) facet of $Ti_3C_2T_x$, and the Ti 2*p* XPS spectrum could be deconvoluted into Ti−C, Ti^{2+}, Ti^{3+}, and Ti^{4+} components, similar with pure $Ti_3C_2T_x$ substrate (Supplementary Fig. 10d).

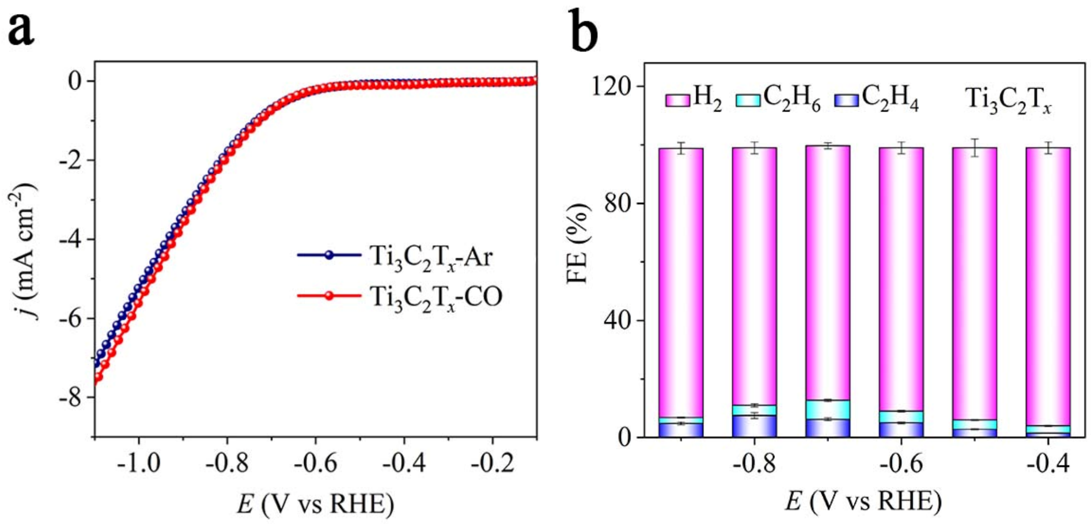

Supplementary Fig. 34. Electrochemical CO reduction performances of pristine $Ti_3C_2T_x$. (a) LSV curves measured in CO- or Ar-saturated 1 M KOH solutions at a scan rate of 10 mV s^{-1}. (b) Faradaic efficiencies (FEs) toward H_2 and CO reduction products, the electrolysis was carried out at each constant potential for 2 h. The error bars correspond to the standard deviations of measurements over three separately prepared samples under the same testing conditions. The FE values of the CO reduction products are much lower than those of Cu-SA/$Ti_3C_2T_x$.

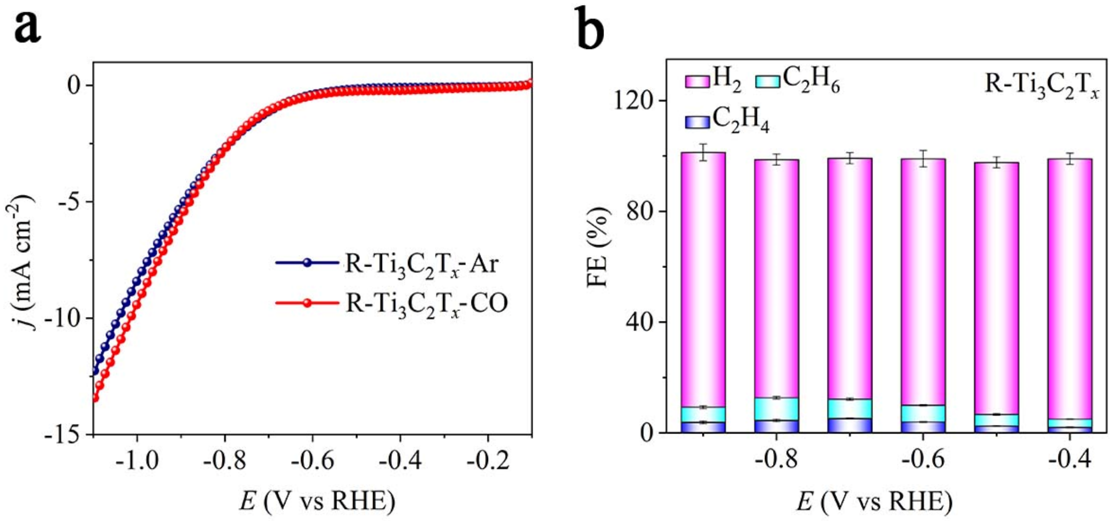

Supplementary Fig. 35. Electrochemical CO reduction performances of R-$Ti_3C_2T_x$. (a) LSV curves measured in CO- or Ar-saturated 1 M KOH solutions at a scan rate of 10 mV s^{-1}. (b) Faradaic efficiencies (FEs) toward H_2 and COR products, the electrolysis was carried out at each constant potential for 2 h. The error bars correspond to the standard deviations of measurements over three separately prepared samples under the same testing conditions. The FE values of the CO reduction product are much lower than those of Cu-SA/$Ti_3C_2T_x$.

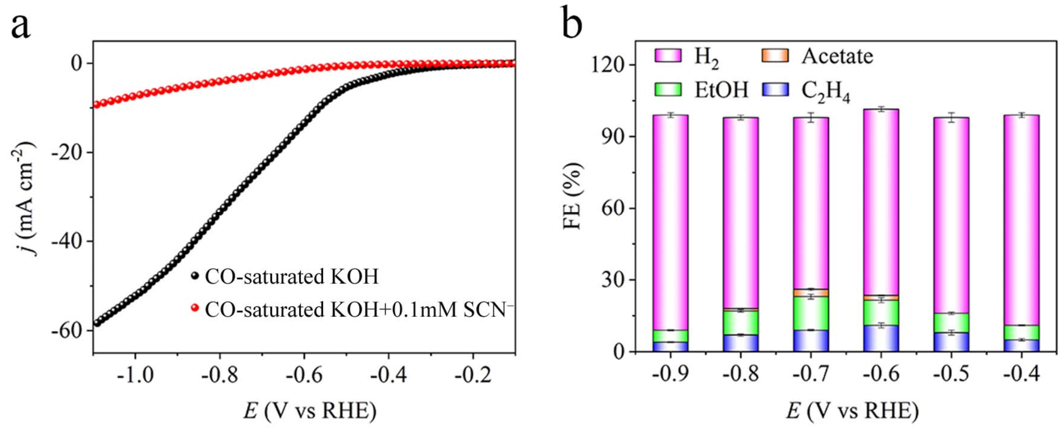

Supplementary Fig. 36. SCN^- poisoning in CO-saturated 1 M KOH. (a) LSV curves of Cu-SA/$Ti_3C_2T_x$ in different electrolyte solutions, of which the black line is the same one as the red one in Fig. 2a. (b) Faradaic efficiencies (FEs) of Cu-SA/$Ti_3C_2T_x$ in CO-saturated 1 M KOH + 0.1 mM KSCN at different working potentials. The error bars correspond to the standard deviations of measurements over three separately prepared samples under the same testing conditions.

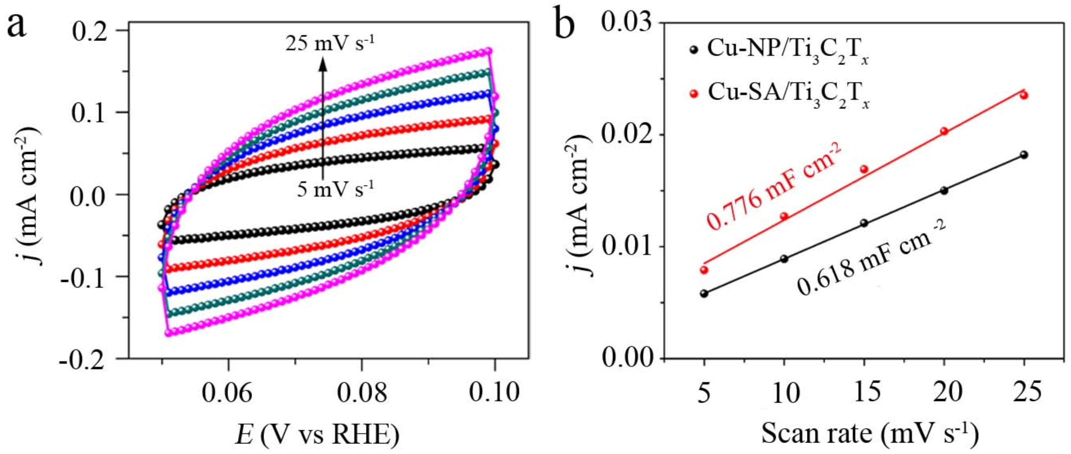

Supplementary Fig. 37. Determination of electrochemical active surface area (ECSA). (a) Cyclic voltammetry curves of Cu-SA/$Ti_3C_2T_x$ in the non-Faradaic capacitance current range at scan rates of 5, 10, 15, 20 and 25 mV s^{-1}. (b) Charging current density differences plotted against scan rates. The ECSAs of CO reduction electrodes were checked by measuring the electrochemical double-layer capacitance (C_{dl}) with cyclic voltammetry. As seen, Cu-SA/$Ti_3C_2T_x$ exhibits a higher C_{dl} of 0.776 mF cm^{-2} in comparison with Cu-NP/$Ti_3C_2T_x$, suggesting more active sites in Cu-SA/$Ti_3C_2T_x$.

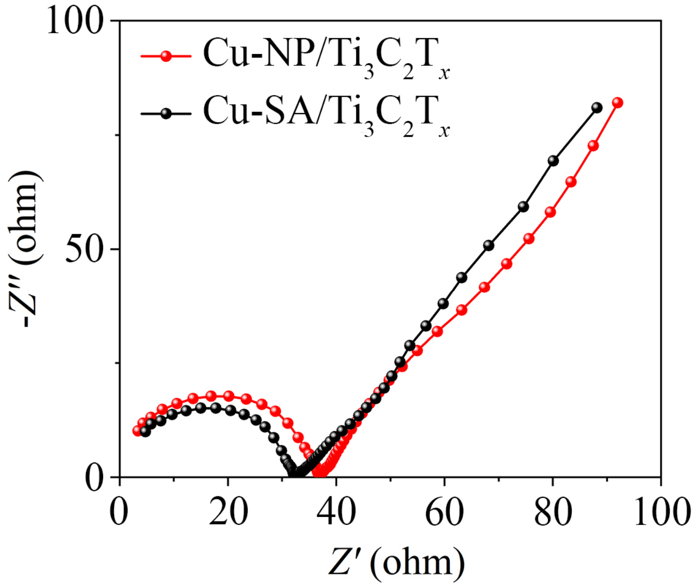

Supplementary Fig. 38. Nyquist plots obtained by electrochemical impedance spectroscopy for Cu-SA/$Ti_3C_2T_x$. Clearly, it indicates that the charge-transfer resistance of Cu-SA/$Ti_3C_2T_x$ is lower than that of Cu-NP/$Ti_3C_2T_x$, suggesting that Cu-SA/$Ti_3C_2T_x$ has a faster charge-transfer capacity for CO reduction.

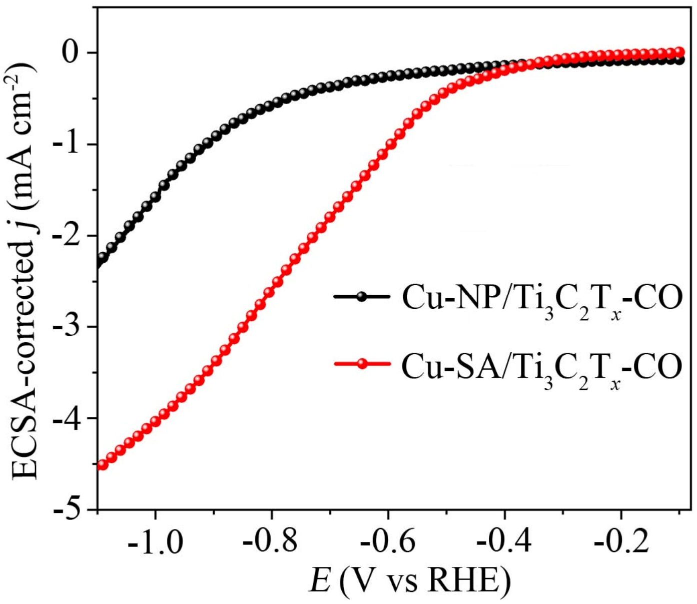

Supplementary Fig. 39. Electrochemical active surface area (ECSA)-corrected current densities vs. applied potentials. Cu-SA/$Ti_3C_2T_x$ exhibited much higher current densities compared with that of Cu-NP/$Ti_3C_2T_x$ after corrected by ECSA.

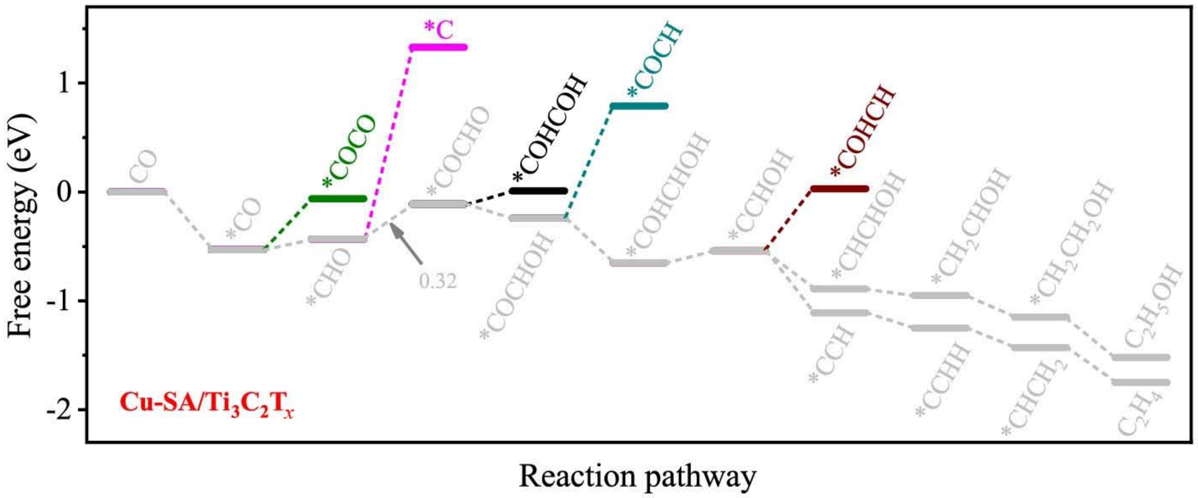

Supplementary Fig. 40. Other possible intermediates of CO reduction on Cu-SA/$Ti_3C_2T_x$.

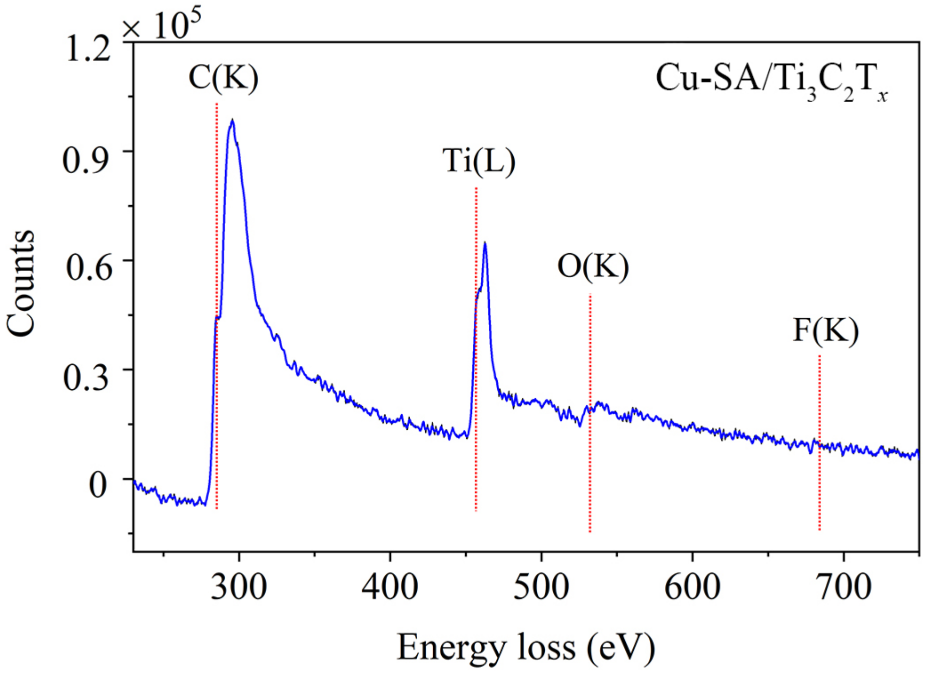

Supplementary Fig. 41. The EELS spectrum of Cu-SA/$Ti_3C_2T_x$. Signals of C K-edge, O K-edge, F K-edge, and Ti L-edge can be observed[9].

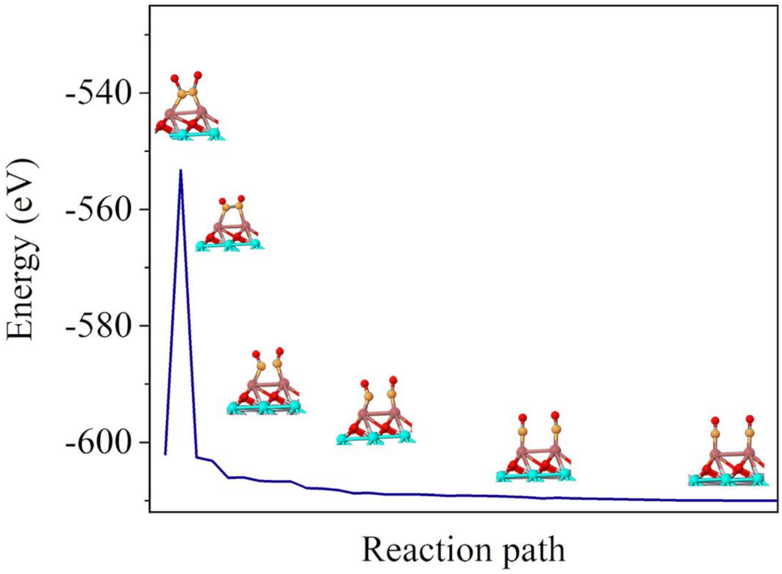

Supplementary Fig. 42. The optimization process of *COCO on Cu-SA/$Ti_3C_2T_x$. A model of 2Cu/$Ti_3C_2T_x$ was used to calculate the 2*CO → *COCO process on Cu-SA/$Ti_3C_2T_x$. For 2Cu/$Ti_3C_2T_x$, two nearest isolated Cu atoms were constructed on $Ti_3C_2T_x$ substrate. The distance between the two Cu atoms is measured to be 3.2 Å. Note that, it is nearer than the actual average Cu–Cu interatomic distance (6.1 Å) in Cu-SA/$Ti_3C_2T_x$ (see details in Supplementary Fig. 7), and this 2Cu/$Ti_3C_2T_x$ model was just used for investigating the possibility of 2*CO coupling on Cu-SA/$Ti_3C_2T_x$. Accordingly, the geometric optimization was proceeded on *COCO-2Cu/$Ti_3C_2T_x$ model to investigate the existence of *COCO intermediate. Unfortunately, the energy minimum value point for *COCO intermediate was not found in the local region. Instead, *COCO was separated to form two isolated *CO. So the 2*CO → *COCO pathway is not applicable on the 2Cu/$Ti_3C_2T_x$ surface.

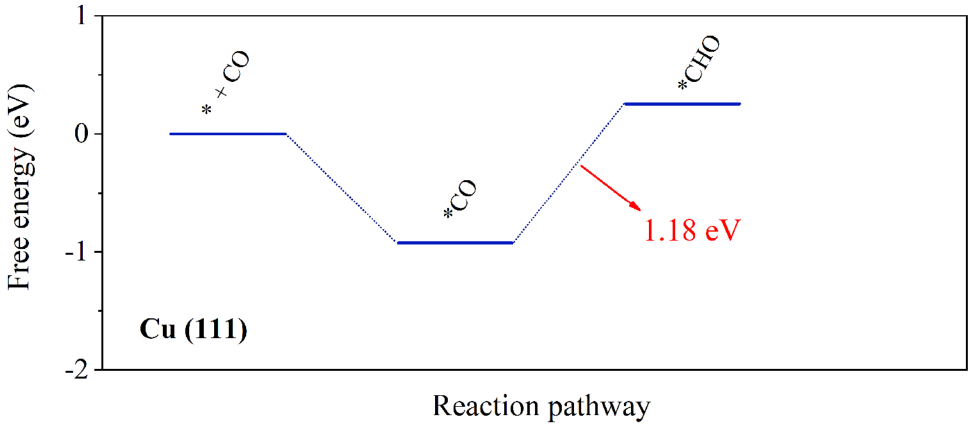

Supplementary Fig. 43. The reaction mechanism of the CO reduction on Cu (111) through *CHO pathway.

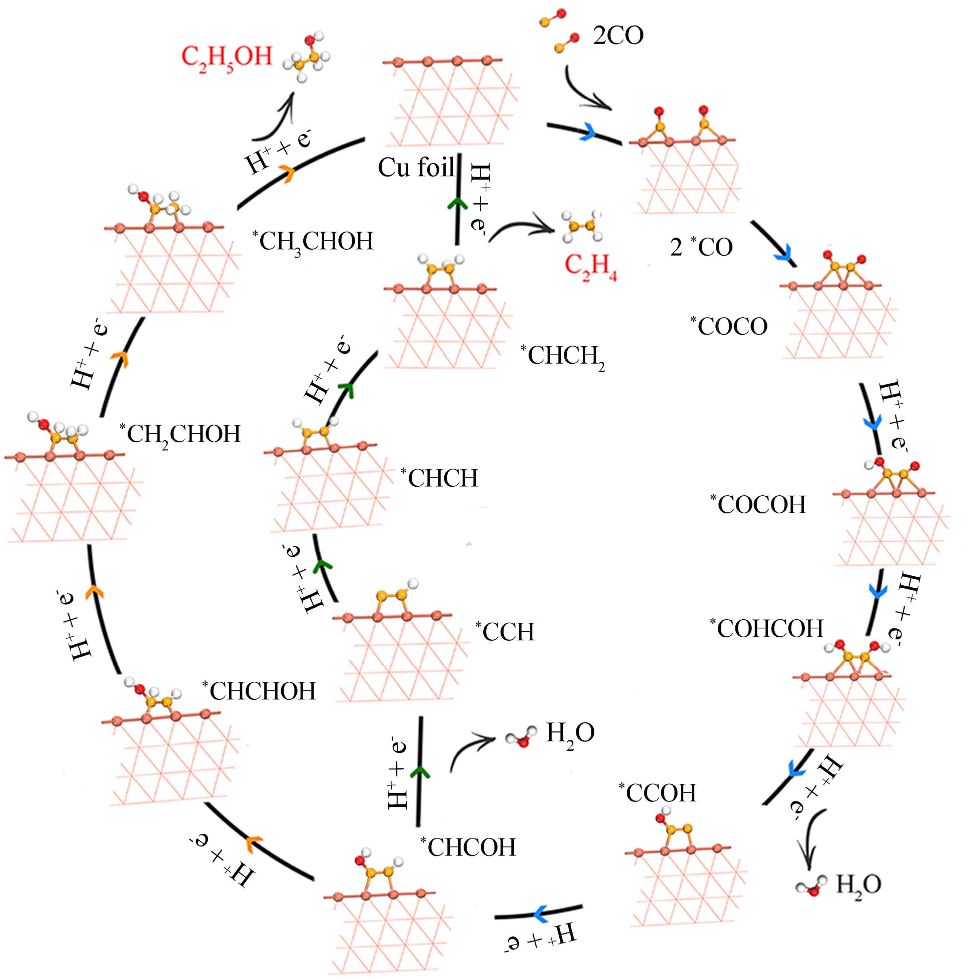

Supplementary Fig. 44. Schematic representations of CO reduction reaction pathways on Cu (111). The pinkish-orange, dark yellow, red and white balls represent Cu, C, O, and H atoms, respectively.

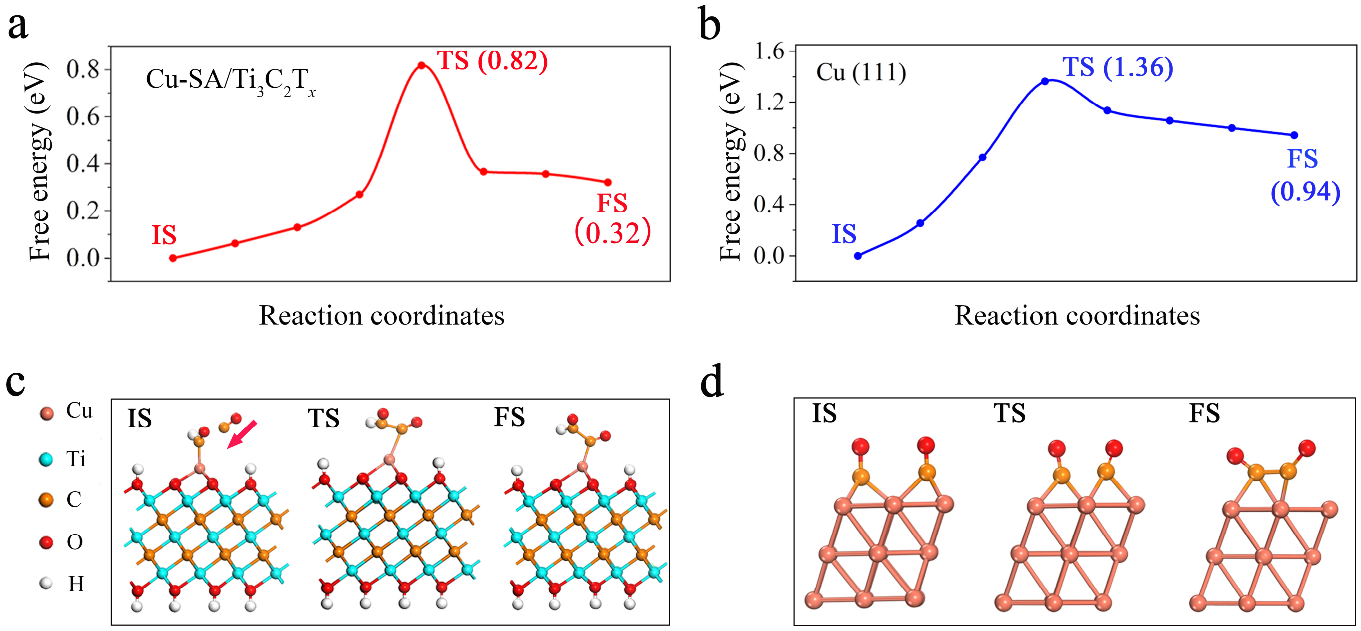

Supplementary Fig. 45. The energy barriers of the rate-determining steps on Cu-SA/$Ti_3C_2T_x$ and Cu (111) surface. (a, b) The energy profiles of the C–C coupling process on the Cu-SA/$Ti_3C_2T_x$ surface (a) and the Cu (111) surface (b). (c, d) The corresponding initial state (IS), transition state (TS) and final state structures (FS) from *CO to *CO-CHO on Cu-SA/$Ti_3C_2T_x$ (c) and from 2*CO to *CO-CO on Cu (111) (d).

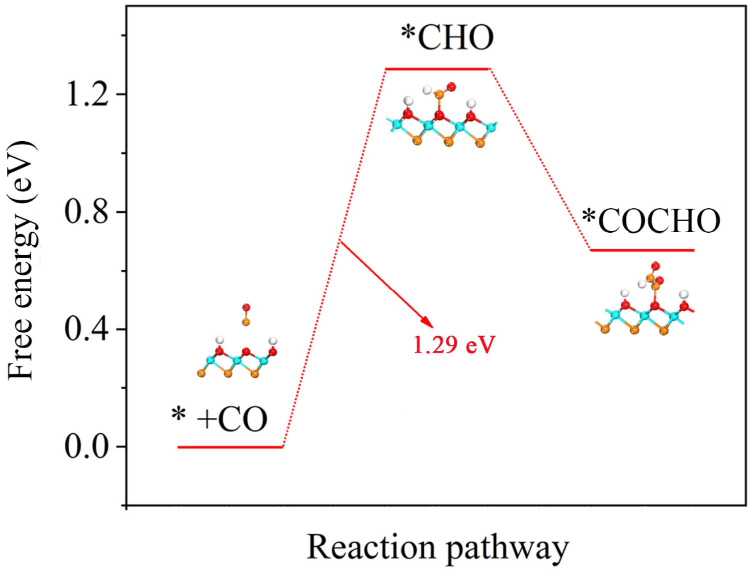

Supplementary Fig. 46. The optimized CO reduction pathway on pure $Ti_3C_2T_x$ surface. Note that the 2*CO → *COCO pathway is infeasible on Cu-SA/$Ti_3C_2T_x$ model as confirmed by the DFT-calculations.

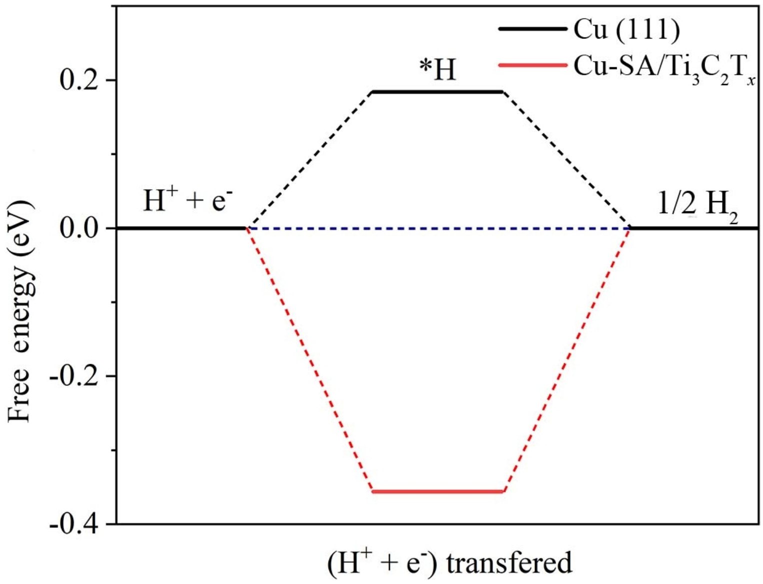

Supplementary Fig. 47. The calculated free-energy diagram of hydrogen evolution reaction catalyzed by Cu (111) and Cu-SA/$Ti_3C_2T_x$.

Supplementary Tables

Supplementary Table 1. Structural parameters extracted from the Cu K-edge EXAFS fitting for Cu-SA/$Ti_3C_2T_x$. (S_0^2 = 0.81)

Sample	Main shell	Atom	N	R (Å)	σ^2 (Å^2)	ΔE_0 (eV)	R factor
**Cu-SA/$Ti_3C_2T_x$**	1st	O	3.2±0.4	2.03	0.01	4.2±0.9	0.01
	2st	Ti	3.3±1.2	3.05	0.02	4.2±0.9	0.01

S_0^2, the amplitude reduction factor derived from fitting the Cu foil; N, the coordination number; R, interatomic distance; σ^2, Debye-Waller factor; ΔE_0, edge-energy shift (accounts for the difference between the zero kinetic energy value of the sample and that of the theoretical model). R factor, the goodness of the fitting. The fitting window is from 1.25–3.0 here.

Supplementary Table 2. Reported catalysts for CO reduction in aqueous electrolytes.

Catalysts	Electrolyte	[a]FE_{C2+} (%)	Products	[b]J_{red}	Stability	Cell	Ref.
**Oxide-derived Cu**	0.1 M KOH	57%@−0.3 V	@−0.3 V EtOH (~42%) Acetate (~15%)	~0.02 mA cm^{-2}@−0.3 V	7 h	Two-compartment cell	*Nature* **2014**, *508,* 504−507
**Cu NPs (grain boundaries)**	0.1 M KOH	~70%@−0.3 V	@−0.3 V EtOH (~35%) Acetate (~35%)	~0.04 mA cm^{-2}@−0.3 V	14 h	Two-compartment cell	*ACS Cent. Sci.* **2016**, *2*, 169−174
**Cu Nanowires**	0.1 M KOH	65%@−0.3 V	@−0.3 V EtOH (~50%) Acetate (~15%)	~0.43 mA cm^{-2}@−0.45 V	1 h	Gas-tight electrolysis cell	*ACS Catal.* **2017**, *7*, 4467−4472
**Polycrystalline Cu**	0.1 M KOH	~42%@−0.59 V	@−0.59 V C_2H_4 (30%) EtOH (~12%)	1.2 mA cm^{-2}@−0.59 V	30 min	Two-compartment cell	*ACS Energy Lett.* **2018**, *3*, 634−640
**Cu NPs**	10 M KOH (−15 °C)	−	@−0.74 V C_2H_4 (17%)	50.8 mA cm^{-2}@−0.85 V	2 h	Gas diffusion electrodes (GDEs)	*ACS Energy Lett.* **2018**, *3*, 855−860

Catalysts	Electrolyte	$^{a}FE_{C2+}$ (%)	Products	$^{b}J_{red}$	Stability	Cell	Ref.
**Cu NPs**	2.0 M KOH	~91%@−0.65 V	@−0.65 V C_2H_4 + Acetate + EtOH	630 mA cm^{-2}@−0.65 V	2 h	Flow cell	*Nat. Catal.* **2018**, *1*, 748−755
**Highly fragmented Cu**	1 M KOH	~57%@−0.66 V	@−0.66 V C_2H_4 (~40%) EtOH (17%) n-Propanol (~19%)	8.5 mA cm^{-2} for n-propanol @−0.45 V	3.3 h	Flow cell	*Nat. Catal.* **2019**, *2*，251−258
**Polycrystalline Cu/PTEE**	0.1 M KOH	65%@−0.94 V	@−0.76 V C_2H_4 (~35%) EtOH (~15%) Acetate (~15%) Methane (~15%)	~150 mA cm^{-2}@−0.94 V	2 h	Flow cell	*ACS Catal.* **2019**, *9*, 4709−4718
**Cu nanosheets**	2 M KOH	~70% @−0.75 V	@−0.75 V C_2H_4 (~15%) Acetate (~48%)	131 mA cm^{-2} for Acetate @−0.75 V	3 h	Flow-cell	*Nat. Catal.* **2019**, *2*，423−430

Catalysts	Electrolyte	$^{a}FE_{C2+}$ (%)	Products	$^{b}J_{red}$	Stability	Cell	Ref.
**Cu NPs**	1 M KOH	~62%@−0.7 V	@−0.7 V<br>C_2H_4 (52.7%)<br>EtOH (~9%)	14.9<br>mA cm^{-2}@−0.7 V	24 h	Two-compartment cell	*Angew. Chem. Int. Edit.* **2020**, *132*, 160−166.
**Polycrystalline Cu**	0.1 M KOH	~60%@−0.63 V	@~−0.63 V<br>C_2H_4 (~40 %)<br>EtOH<br>Acetate	-	-	Custom-designed electrochemical cell	*ACS Catal.* **2018**, *8*, 7445−7454.
**Cu nanoflower electrodes**	0.1 M KOH	~100%@~−0.23 V	@~−0.23 V<br>CH_3CHO (~60%)<br>Acetate (~20%)<br>EtOH (~17)	~−0.25<br>mA cm^{-2}@~−0.23 V	10 h	Two-compartment cell	*Nat. Catal.* **2019**, *2*, 702−708.
**Cu-SA/$Ti_3C_2T_x$**	1 M KOH	~98%@−0.7 V	@−0.7 V<br>C_2H_4 (71 %)<br>EtOH (25 %)<br>Acetate (2%)	~22.1<br>mA cm^{-2}@−0.7 V	68 h	Two-compartment cell	***This work***

[a] FE_{C2+} means the overall Faradaic efficiency of C_{2+} products;

$^{b}J_{red}$ means the current density of the reduction products.

Supplementary Table 3. Contributions to the free energies of adsorbed intermediates on Cu-SA/$Ti_3C_2T_x$ from zero-point energy (ZPE) correction, enthalpic temperature correction, and entropy contribution. The * refers to a binding site, and all are given in eV.

Adsorbant Species	ZPE	$\int C_p dT$	Entropy S
*CO	0.202340	0.07512	0.000543
*CHO	0.437156	0.07273	0.000555
*COCHO	0.680410	0.13405	0.001001
*COCHOH	0.995937	0.11405	0.000826
*COHCHOH	1.273490	0.1486	0.001009
*CCHOH	0.896533	0.12883	0.000912
*CCH	0.477784	0.08553	0.000603
*CCHH	0.734807	0.09926	0.000686
*$CHCH_2$	1.044965	0.08586	0.000599
CH_2CH_2	1.366320	0.12022	0.001000
*CHCHOH	1.222207	0.12686	0.000919
*CH_2CHOH	1.544703	0.10857	0.000755
*CH_2CH_2OH	1.832620	0.11427	0.000783
C_2H_5OH	2.119223	0.13865	0.001046

Supplementary Table 4. Contributions to the free energies of adsorbed intermediates on Cu (111) from zero-point energy (ZPE) correction, enthalpic temperature correction, and entropy contribution. The * refers to a binding site, and all are given in eV.

Adsorption species	ZPE	$\int C_p dT$	Entropy S
*CO	0.175907	0.05447	0.000317
*COCO	0.368749	0.116994	0.000467
*COCOH	0.62145	0.15995	0.000671
*COHCOH	0.97073	0.1309	0.000855
*CCOH	0.844724	0.15632	0.000615
*CHCOH	0.94896	0.15346	0.000791
*CCH	0.61655	0.17861	0.000561
*CHCH	0.67839	0.15995	0.000311
*$CHCH_2$	1.0714	0.16391	0.001161
CH_2CH_2	1.36632	0.12022	0.001000
*CHCHOH	1.24293	0.18538	0.001334
*CH_2CHOH	1.79487	0.14426	0.001015
*CH_3CHOH	1.95329	0.14997	0.001035
C_2H_5OH	2.11922	0.13865	0.001046

Supplementary References

1 Poulston, S., Parlett, P., Stone, P. & Bowker, M. Surface oxidation and reduction of CuO and Cu_2O studied using XPS and XAES. *Surf. Interface Anal.* **24**, 811–820 (1996).

2 Halim, J., Cook, K. M., Naguib, M., Eklund, P., Gogotsi, Y., Rosen, J. & Barsoum, M. W. X-ray photoelectron spectroscopy of select multi-layered transition metal carbides (MXenes). *Appl. Surf. Sci.* **362**, 406–417 (2016).

3 Naguib, M., Kurtoglu, M., Presser, V., Lu, J., Niu, J., Heon, M., Hultman, L., Gogotsi, Y. & Barsoum, M. W. Two-dimensional nanocrystals produced by exfoliation of Ti_3AlC_2. *Adv. Mater.* **23**, 4248–4253 (2011).

4 Wan, Q., Wei, F., Wang, Y., Wang, F., Zhou, L., Lin, S., Xie, D. & Guo, H. Single atom detachment from Cu clusters, and diffusion and trapping on CeO_2(111): implications in Ostwald ripening and atomic redispersion. *Nanoscale* **10**, 17893–17901 (2018).

5 Dvořák, F., Farnesi Camellone, M., Tovt, A., Tran, A., Negreiros, F. R., Vorokhta, M., Skála, T., Matolínová, I., Mysliveček, J., Matolín, M. & Fabris, S. Creating single-atom Pt-ceria catalysts by surface step decoration. *Nat. Commun.* **7**, 10801 (2016).

6 Su, Y.-Q., Liu, J.-X., Filot, I. A. & Hensen, E. J. Theoretical study of ripening mechanisms of Pd clusters on ceria. *Chem. Mater.* **29**, 9456–9462 (2017).

7 Crowley S. & Castaldi M. J. Mechanistic insights into catalytic ethanol steam reforming using isotope-labeled reactants. *Angew. Chem. Int. Ed.* **55**, 10650 –10655 (2016).

8 Lum, Y. & Ager, J. W. Evidence for product-specific active sites on oxidederived Cu catalysts for electrochemical CO_2 reduction. *Nat. Catal.* **2**, 86–93 (2019).

9 Sang, X., Xie, Y., Lin, M.-W., Alhabeb, M., Van Aken, K. L., Gogotsi, Y., Kent, P. R. C., Xiao, K. & Unocic, R. R. Atomic defects in monolayer titanium carbide ($Ti_3C_2T_x$) MXene. *ACS Nano* **10**, 9193–9200 (2016).
